# *GUN* Mutants: New Weapons To Unravel Ascospore Germination Regulation in the Model Fungus *Podospora anserina*

**DOI:** 10.1128/spectrum.01461-22

**Published:** 2023-02-14

**Authors:** Alexander Demoor, Isabelle Lacaze, Roselyne Ferrari, Christophe Lalanne, Philippe Silar, Sylvain Brun

**Affiliations:** a Université Paris Cité, Laboratoire Interdisciplinaire des Energies de Demain/UMR 8236, Paris, France; Centro de Investigaciones Biologicas CSIC

**Keywords:** mitochondria, *Podospora anserina*, *Fungi*, carnitine-acetyl transferase, appressorium, ascospore germination, filamentous fungi, peroxisomes

## Abstract

In Podospora anserina as in many other *Ascomycetes*, ascospore germination is a regulated process that requires the breaking of dormancy. Despite its importance in survival and dispersal, ascospore germination in filamentous fungi has been poorly investigated, and little is known about its regulation and genetic control. We have designed a positive genetic screen that led to the isolation of mutants showing uncontrolled germination, the *GUN* (*Germination UNcontrolled*) mutants. Here, we report on the characterization of the *gun1^SG^* (*Spontaneous Germination*) mutant. We show that *gun1^SG^* is mutated in *Pa_6_1340*, the ortholog of Magnaporthe oryzae *Pth2*, which encodes a carnitine-acetyltransferase (CAT) involved in the shuttling of acetyl coenzyme A between peroxisomes and mitochondria and which is required for appressorium development. Bioinformatic analysis revealed that the mutated residue (I441) is highly conserved among *Fungi* and that the mutation has a deleterious impact on the protein function. We show that GUN1 is essential for ascospore germination and that the protein is localized both in mitochondria and in peroxisomes. Finally, epistasis studies allowed us to place *GUN1* together with the *PaMpk2* MAPK pathway upstream of the *PaNox2*/*PaPls1* complex in the regulation of ascospore germination. In addition, we show that *GUN1* plays a role in appressorium functioning. The pivotal role of *GUN1*, the ortholog of *Pth2*, in ascospore germination and in appressorium functioning reinforces the idea of a common genetic regulation governing both appressorium development and melanized ascospore germination. Furthermore, we characterize the second CAT encoded in *P. anserina* genome, *Pa_3_7660*/*GUP1*, and we show that the function of both CATs is conserved *in P. anserina*.

**IMPORTANCE** The regulation of ascospore germination in filamentous fungi has been poorly investigated so far. To unravel new genes involved in this regulation pathway, we conducted a genetic screen in *Podospora anserina*, and we isolated 57 mutants affected in ascospore germination. Here, we describe the *Germination UNcontrolled One* (*gun1^SG^*) mutant, and we characterize the gene affected. GUN1 is a peroxisomal/mitochondrial carnitine-acetyltransferase required for acetyl coenzyme A shuttling between both organelles, and we show that *GUN1* is a pleiotropic gene also involved in appressorium functioning similarly to its ortholog, the pathogenesis factor *Pth2*, in the plant pathogen Magnaporthe oryzae. Given the similarities in the regulation of appressorium development and ascospore germination, we speculate that discovering new genes controlling ascospore germination in *P. anserina* may lead to the discovery of new pathogenesis factors in pathogenic fungi. The characterization of *GUN1*, the ortholog of M. oryzae *Pth2*, represents a proof of concept.

## INTRODUCTION

Fungi are eukaryotic microorganisms able to resist adverse environments and disseminate through the formation of asexual (mitospores) or sexual spores (meiospores). Representing the final product of sexual reproduction, meiospores ensure both survival and dissemination and carry genetic diversity, allowing them to adapt in changing environments (1). Spore germination is a crucial step in the fungal life cycle, and its successful completion is thus essential for survival and spread. Conidial germination represents the starting point of infection for important pathogenic species such as Aspergillus fumigatus or Magnaporthe oryzae, and its regulation has been widely studied (2, 3). In contrast, little is known about the regulation of ascospore germination, although ascospores are the key dispersal propagule for many other important pathogenic species such as *Leptosphaeria maculans*, the main pathogen for oilseed rape cultures (4, 5). Although conidial germination and ascospore germination share some morphological similarities, several studies have demonstrated that their regulation is different (6). Podospora anserina, a saprotrophic coprophilous *Ascomycete* from the *Sordariales* order, emerges as an excellent genetic model system to study ascospore germination. *P. anserina* produces asexual spermatia that are unable to germinate, as well as ascospores that represent the only germinating spores in this fungus. This species forms after fertilization pear-shaped fruiting bodies called perithecia, each harboring a few hundred to a thousand ascospores differentiated in asci. This pseudohomothallic fungus produces a minority of homokaryotic ascospores of either the *mat*+ or the *mat*– mating type (heterothallic lifestyle) and a majority of heterokaryotic self-fertile *mat*+/*mat*– ascospores, allowing pseudohomothallic lifestyle (7). The melanized ascospores of *P. anserina* are in a dormant state and require a stimulus in order to germinate. *P. anserina* is a coprophilous fungus reported to grow preferentially on herbivores’ dung. Indeed, in the wild, the breaking of dormancy of *P. anserina* ascospores is usually triggered by the passage through herbivores digestive tract, leading to germination on dung. This stimulus is reproduced in laboratory conditions by placing the ascospores on a germination medium composed of ammonium acetate and Bacto peptone (7). So far, the respective roles of acetate, ammonium, and Bacto peptone in triggering germination have not been determined. It is worth noting that yeast extract is often added to the germination medium to increase germination rate, but here again, its role in triggering germination remains unknown. In *P. anserina*, ascospore germination takes place in several steps: (i) the activation by the stimulus (dormancy breaking), (ii) the formation of the germination pore at the apex of the spore (opposite to the primary appendage), and (iii) the extrusion of a germination peg/bubble from which one or several (often two) germinating hyphae emerge (see Movie S1).

It was shown that both H_2_O_2_ and O_2_^–^ reactive oxygen species (ROS) are produced during ascospore germination in *P. anserina*, suggesting that ROS play an important role at this stage (8). Knockout of the superoxide (O_2_**^−^**)-producing enzyme NADPH oxidase *PaNox2* encoding gene and of the *PaPls1* tetraspanin encoding gene, as well as the regulatory subunit *PaNoxR*, leads to an almost complete abolishment of ascospore germination (8–10). The mutations (or deletions) of the orthologs of *PaNox2* and *PaNoxR* genes lead to a loss of ascospore germination ability in both model species Sordaria macrospora and Neurospora crassa, highlighting the conservation of Nox2/B, and NoxR functions in the regulation of ascospore germination (11, 12). Strikingly, the NADPH oxidase complexes Nox1/A, Nox2/B, NoxR, and Pls1 are required for appressorium functioning in the plant-pathogenic fungus M. oryzae (10, 13, 14). Considering that the ascospores in *P. anserina* and the appressorium in M. oryzae are both melanized structures, we have hypothesized that similar components and, in particular, the Nox2/B-Pls1-NoxR complex regulate cellular processes such as the formation of the pore through which arise the penetration peg in M. oryzae and the germination peg in *P. anserina* (15, 16).

The same hypothesis applies to the mitogen-activated protein kinase (MAPK) pathway *FUS3/PMK1/PaMpk2* (S. cerevisiae/M. oryzae/P. anserina) involved in appressorium development in M. oryzae, as well as in ascospore germination in *P. anserina* (17–19). We have shown that loss of function of each of the three kinases of the MAPK cascade in *P. anserina*, i.e., *PaTlk2*, *PaMKK2*, and *PaMpk2*, abolishes ascospore germination. Conversely, the activation of the pathway in the constitutively active *PaMKK2^c^* mutant leads to the spontaneous germination of ascospores (17). In N. crassa, mutants of MAK-2, the ortholog of FUS3/PaMpk2, and mutants of pp-1/STE12, the downstream transcription factor of the cascade, show an almost complete lack of ascospore germination (20, 21). In addition, knockout of the *STE12* ortholog in *S. macrospora* also impairs ascospore germination (12, 22). Interestingly, these three species harbor melanized ascospores that require a stimulus to germinate. This raises questions regarding the role of this pigment during spore germination.

As in other fungi, melanin in *P. anserina* is synthesized through an enzymatic cascade, starting with the PaPKS1 polyketide synthase. Inactivation of this key enzyme results in total inability to produce melanin in hyphae, perithecia, and ascospores, as observed in the *Papks1^193^* and the *Δpapks1* null mutants (23–25). Remarkably, nonmelanized ascospores in the *Papks1^193^* mutant escape dormancy and germinate spontaneously. Since the presence of melanin in cell wall contributes to its rigidity (26), it is assumed that lack of melanin weakens the ascospore cell wall, leading to “accidental,” uncontrolled germination. In line with this, culturing and crossing *P. anserina* on medium containing the fungicide tricyclazole, an inhibitor of melanin production in fungi, allows ascospores bearing mutations in genes essential for germination, such as *PaNox2*, *PaNoxR*, *PaPls1*, or *PaMpk2*, to spontaneously germinate (8–10, 17, 23). It should be highlighted that these nonmelanized ascospores are fragile and lose viability if manipulated (when moved with a needle for harvesting for instance).

A defect in ascospore melanization and germination is also observed in peroxisome biogenesis, peroxisomal β-oxidation, and mitochondrial β-oxidation mutants. In these mutants, depletion of acetyl coenzyme A (acetyl-CoA), the direct product of β-oxidation and the main precursor for melanin biosynthesis, is supposed to account for both defects (27–30). Peroxisomes are organelles of fundamental importance, in particular for fungal pathogenesis (31–33). In M. oryzae, mutants affected in peroxisomal β-oxidation and acetyl-CoA metabolism show defects in appressorium development, appressorium demelanization, and lack of pathogenicity (34, 35). In addition, the carnitine-acetyltransferase (CAT) *Pth2* mutant was isolated in a forward genetic screen designed to uncover pathogenicity mutants in M. oryzae, underlining the central role of acetyl-CoA metabolism during M. oryzae infection (36–38).

Despite the lack of knowledge on ascospore germination, this process has never been subjected to any dedicated genetic screening. Here, we describe the first genetic screening of ascospore germination mutants. Previous observations have shown that a negative genetic screening of mutants defective for ascospore germination (i) is time-consuming compared to positive screening; (ii) may lead to the isolation of mutants nonspecifically affected in any kind of cellular processes, eventually leading to lack of viability/germination of ascospores; and (iii) brings intrinsic issues in genetic analysis, since these nongerminating mutants are blocked in their sexual/life cycle. With that in mind, we have designed a direct genetic screen aiming at isolating spontaneous germination mutants in the wild-type (WT) *P. anserina* strain normally showing controlled germination. Moreover, to isolate mutants of the *PaNox2*/*PaPls1* pathway, we also screened for suppressors of *ΔPaNox2* and *ΔPaPls1* strains, for which germination was restored. We describe here the *Germination UNcontrolled One* (*gun1^SG^*) mutant, and we identify and characterize the gene affected, as well as its paralogue, the “*GUN1 Paralogue One*” gene (*GUP1*). Functional analysis of both genes shows that *GUN1* codes for the ortholog of the M. oryzae *Pth2/Crat1* CAT (*CAT2* in Saccharomyces cerevisiae; *AcuJ* in Aspergillus nidulans), that *GUP1* codes for the ortholog of the FacC CAT in A. nidulans and *Crat2* in M. oryzae (36, 37, 39–41), and that only GUN1 is important for ascospore germination and melanization.

## RESULTS

### Isolation of germination uncontrolled-GUN mutants.

**FIG 1:**
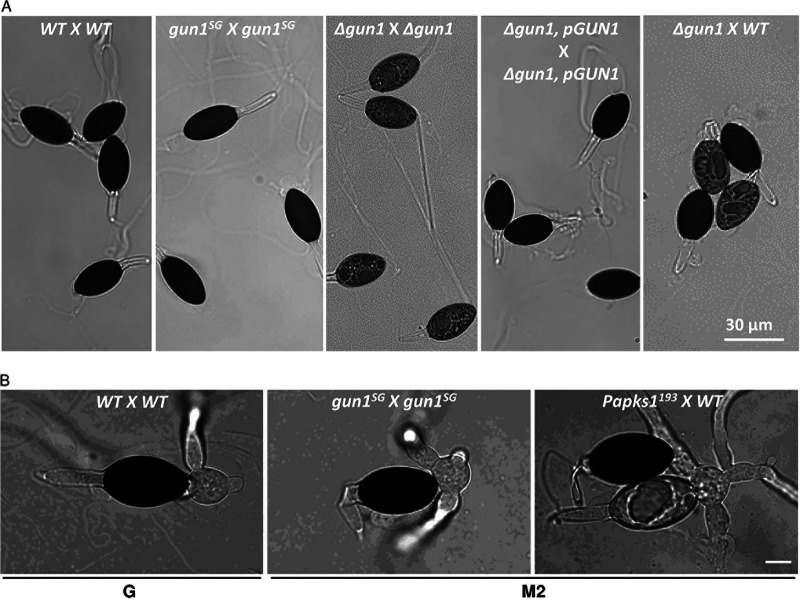
Heterokaryotic ascospores morphology and germination. (A) Ascospore morphology. The crosses performed to obtain the different ascospore genotypes shown are indicated in the picture. *GUN1/GUN1* (WT), *gun1^SG^*/*gun1^SG^*, and *Δgun1 pGUN1/Δgun1 pGUN1* ascospores are fully melanized compared to *Δgun1/Δgun1* ascospores which are partially demelanized. The last row shows FDS ascus composed of two *GUN1/GUN1* (WT) and two *Δgun1/Δgun1* (*Δgun1*) ascospores. The ascospores were mounted in water. Scale bar, 30 μm. (B) Ascospore germination. *GUN1/GUN1* (WT) ascospores require G medium to germinate, while *gun1^SG^*/*gun1^SG^* and *Papks1^193^/Papks1^193^* ascospores germinate in M2 medium (and in G medium [not shown]). Germination occurs through the germination pore located at the tip of the ascospore. (Third column) The melanized ascospore has the *PaPKS1/PaPKS1* genotype, and the nonmelanized ascospore has the *Papks1^193^/Papks1^193^* genotype. Scale bar, 10 μm.

**FIG 2:**
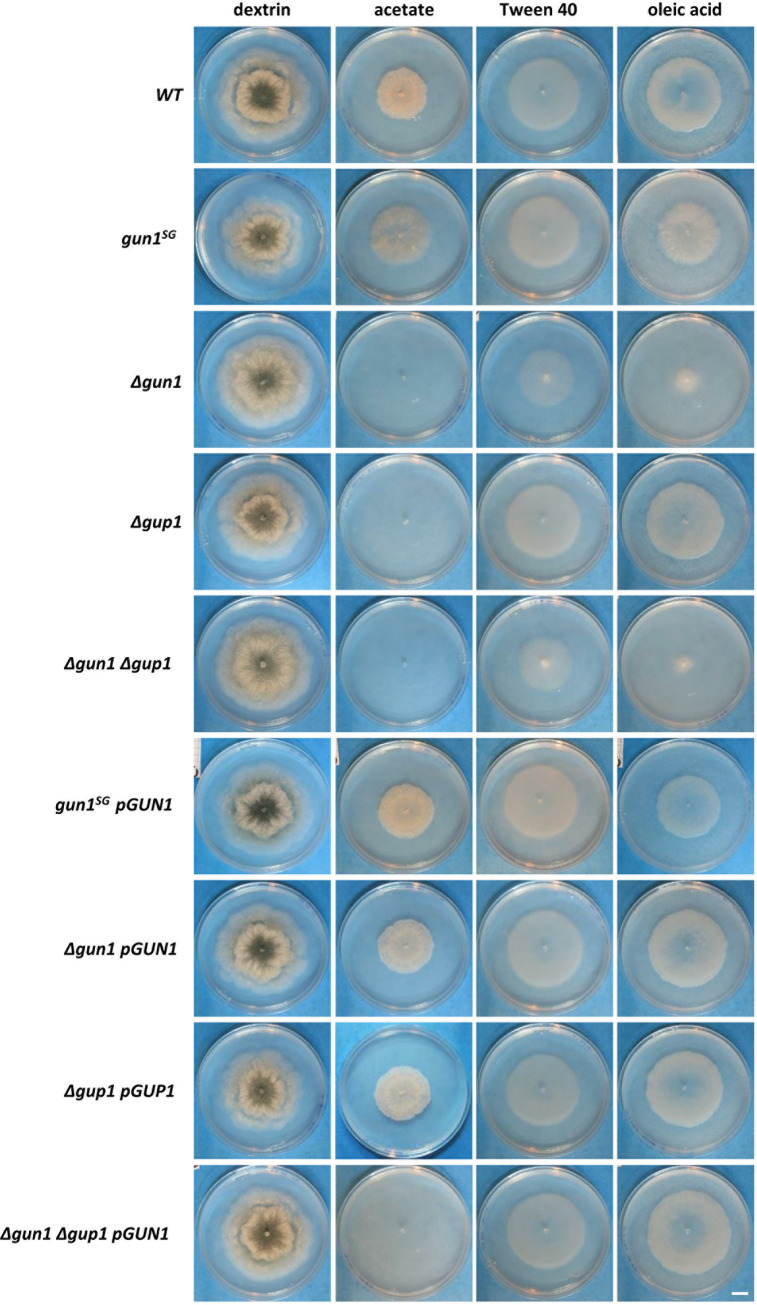
Growth on different carbon sources. Pictures were taken after 4 days of culture. All of the media have same composition, except for the carbon source. The Tween 40 control medium and the oleic acid medium contain 0.5% Tween 40. Scale bar, 1 cm.

As presented above, *P. anserina* ascospores are dormant and do not germinate on standard M2 medium. They require a stimulus to germinate (see Movie S1 in the supplemental material for wild-type germination), provided in laboratory conditions in the specific germination G medium, supplemented with yeast extract (YE) to increase germination rate (42). Rather than screening for mutants unable to germinate, we set up a positive genetic screen allowing isolation of mutants producing spontaneously germinating ascospores on M2 medium. In parallel, we screened for suppressors of the germination defect of the *ΔPaNox2* and *ΔPaPls1* mutant strains (8, 16) using the same protocol. Self-fertile (*mat+/mat–*) mycelia of the *S*, *ΔPaNox2*, and *ΔPaPls1* strains were exposed to UV mutagenesis, and mutants producing spontaneously germinating ascospores on standard M2 medium were isolated (see Fig. S1 and Materials and Methods). Mutant screening allowed recovery of 22 suppressors of the *ΔPaNox2* mutant, 16 suppressors of the *ΔPaPls1* mutant, and 19 mutants from the *S* strain, all of them producing ascospores spontaneously germinating on standard M2 medium (see Table S1). Genetic analysis of these mutants revealed that for all of them, the mutant phenotype was controlled by a single locus. We then checked the germination process in these 57 mutants by microscopic analysis. For all the suppressors of the *ΔPaNox2* and *ΔPaPls1* mutants and 13 *S* strain mutants, ascospore germination was morphologically abnormal. In these mutants, we could observe germination through the primary appendage, the hyaline and elongated structure at the posterior part of the melanized ascospore that gives the name to the genus “*Podo-spora*” (Fig. 1; see also Movie S1). The latter selection was important to possibly discard structural mutants in which the ascospore cell wall was impaired, leading to “accidental” germination or mutants in which the cell constituting the primary appendage failed to degenerate. Indeed, in *P. anserina* the cell constituting the primary appendage degenerates; otherwise, a germ tube arises from this cell, leading to spontaneous germination. Only 6 of the mutants isolated from the wild-type *S* strain showed morphologically “normal” germination proceeding through the germination pore (see Table S1 and Movie S1). We speculated that these six mutants represent mutants of genes involved in the signaling pathway that controls germination and were named *GUNx^SG^* for *Germination Uncontrolledx^Spontaneous Germination^*, where “*x*” stands for the mutant number. Among these mutants, one had the particular characteristic to germinate on agar plates devoid of carbon and nitrate sources, while the five others did not (data not shown). We therefore started the characterization of this mutant that we named the *gun1^SG^* mutant. This mutant differentiated ascospores with a normal shape (Fig. 1A) and with visually normal melanization and spontaneous germination through the germination pore (Fig. 1B). We determined the germination rate of this mutant on M2 medium, as well as on G+YE medium, and compared it to the wild type. Throughout our experiments, we never observed germination of wild-type ascospores when sown on M2 medium. In contrast, when *gun1^SG^/gun1^SG^* heterokaryotic ascospores were transferred onto M2 medium to estimate the germination rate, 54/100 germinated. In the same experiment, 94/100 germinated on G+YE, a rate comparable to wild-type (WT) ascospores (92/100). Once germination was initiated, development of the mycelium produced by the *gun1^SG^* mutant was identical to the wild type: the *gun1^SG^* mutant showed wild-type vegetative growth and mycelium morphology, fertility, ascospore production and appressorium formation. However, despite this apparently normal appressorium formation within cellophane, the *gun1^SG^* mutant was delayed by 1 day compared to the wild type for breaching the cellophane layer (Fig. 2 and Table 1; see also Fig. S2 and S3 in the supplemental material).

**TABLE 1:** Cellophane penetration assay^*a*^

Strain	Day
Wild type	2
*gun1^SG^* mutant	3
*Δgun1* mutant	4
*Δgup1* mutant	2
*Δgun1 Δgup1* mutant	4

a The listed strains were cultured for 5 days on M2 medium topped with a cellophane layer at 27°C. After 2, 3, 4, and 5 days of culture, the cellophane layer was removed to check fungal growth in the medium underneath. The day the cellophane was breached is indicated. Three replicates were made for each day of culture. This experiment was repeated twice.

### *GUN1* encodes a carnitin-acetyltransferase.

**FIG 3:**
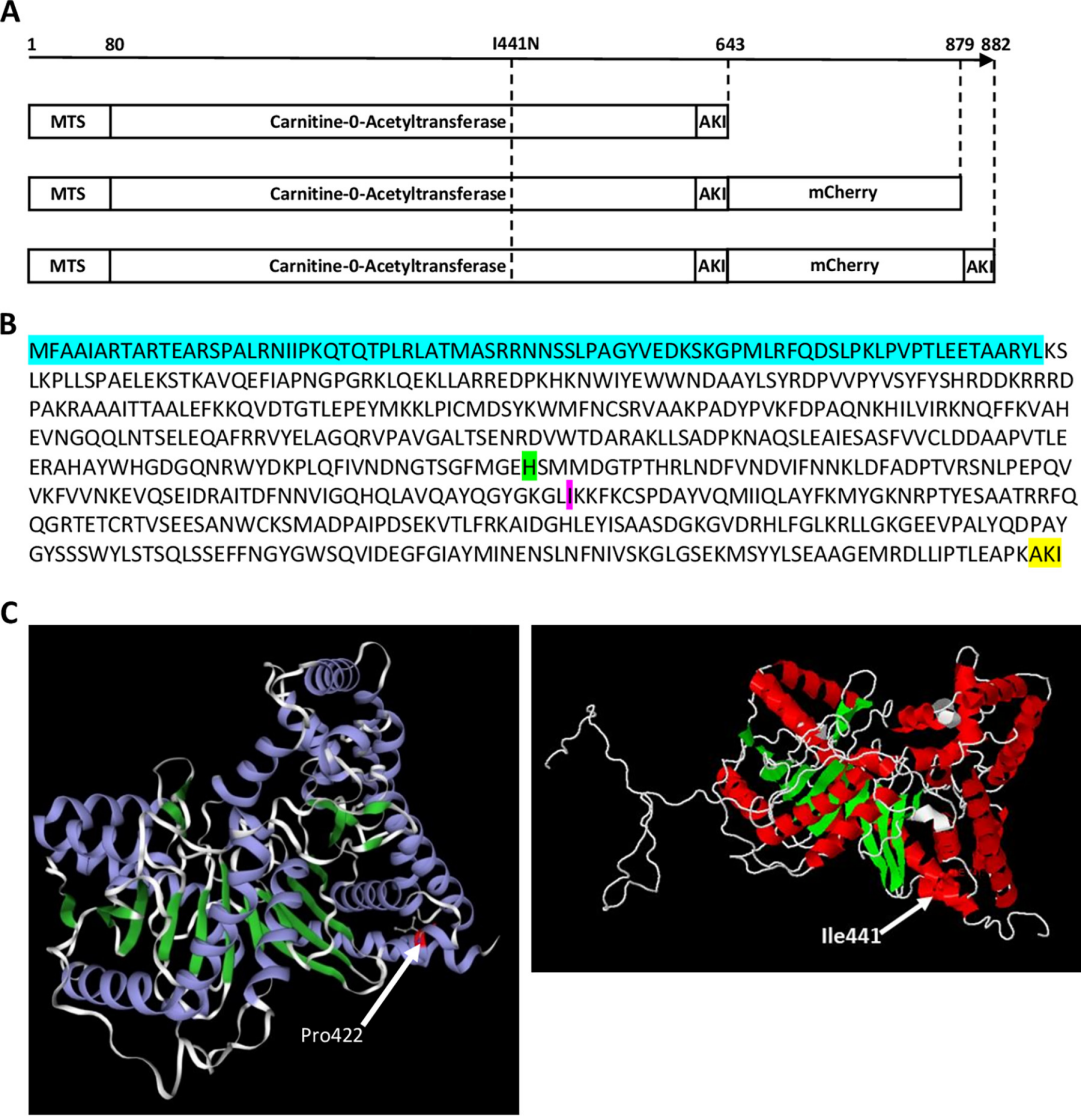
GUN1 and gun1^SG^ protein structures. (A) Schematic representations of GUN1, GUN1-mCherry, and GUN1-mCherry-AKI. MTS, mitochondrial targeting signal. AKI is the GUN1 PTS1-peroxisome targeting sequence. The I441N mutation present in gun1^SG^ is indicated. (B) GUN1 amino acid sequence. The MTS is highlighted in blue, the histidine of the catalytic site is highlighted in green, the I441 is highlighted in magenta, and the AKI PTS1 is highlighted in yellow. (C) 3D structure of murine CAT (PDB 2H3P) (left) and 3D modelization of GUN1 using the I-TASSER modeling tool (right). The isoleucine, I441, in GUN1 and the proline P422 (numbered P396 in the PDB 2H3P crystallized protein) corresponding to the proline aligned to the isoleucine 441 in the murine CAT are indicated by an arrow.

The gene mutated in *gun1^SG^* was identified through whole-genome sequencing. To that end, this mutant was backcrossed five times with the wild-type strain beforehand in order to eliminate most of the mutations generated during UV mutagenesis but not genetically linked to the mutation responsible for the mutant phenotype. The analysis of the *gun1^SG^* whole-genome sequence revealed the presence of six silent mutations, and three missense mutations, one in *Pa_1_13700*, which encodes a putative protein of unknown function, another in *Pa_5_7800*, encoding a putative phosphoketolase, and the last one in the *Pa_6_1340* CoDing Sequence (CDS), where an isoleucine was changed into an asparagine (I441N), caught our attention (Fig. 3). Transcriptome sequencing (RNA-seq) data indicated that *Pa_6_1340* was strongly induced (fold change = 56) during ascospore germination, underlining the involvement of this gene during ascospore germination (A. Demoor, unpublished data). This CDS encodes a putative peroxisomal/mitochondrial carnitine-acetyltransferase (CAT) of 643 amino acids (43–46). Homologs of this gene have previously been studied in S. cerevisiae, Aspergillus nidulans, *Giberella zeae*, Sclerotinia sclerotiorum, and M. oryzae, where they are involved in acetate/acetyl coenzyme A (acetate/acetyl-CoA) metabolism and more particularly in pathogenicity and appressorium development in the phytopathogenic species mentioned (36, 37, 39, 47–49). Regarding the role of acetate and peroxisomes in the control of germination in *P. anserina* and given the induction of *Pa_6_1340* during ascospore germination, this gene emerged as a particularly good candidate for further study.

**FIG 4:**
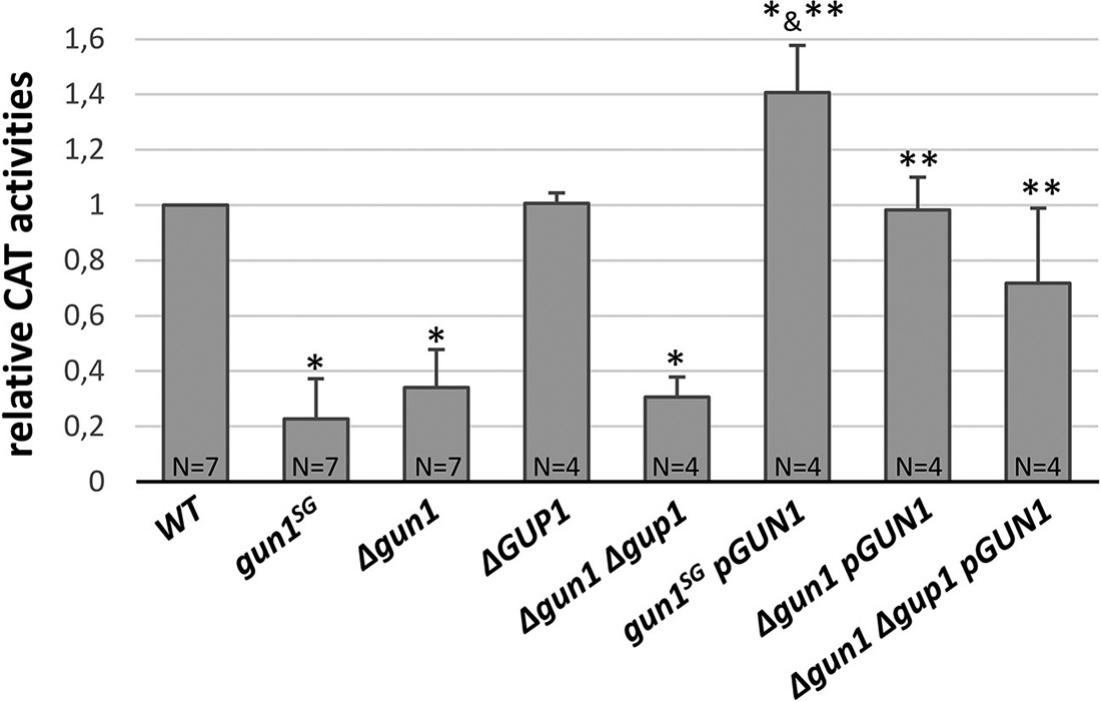
Relative CAT enzymatic activity in mycelial extracts. CAT activity was assayed through the spectrophotometric measure of CoA-SH produced per min per mg of protein in cell extracts in the presence of carnitine. Activities are reported as the activity ratio of the wild-type (WT) *S* strain. The CAT activity means and standard deviations have been calculated based on four to seven biological replicates (*N* is indicated for each genotype). An exact two-sample Fisher-Pitman permutation test was used to compare CAT activities. *, CAT activities significantly different from the WT (*P* < 0.05); **, CAT activities in complemented strains significantly different from the activity in the respective mutant strains (*P* < 0.05).

In order to explore the role of *Pa_6_1340* in the ascospore germination process, a gene replacement was performed, in which the *Pa_6_1340* CDS was substituted by a hygromycin B resistance marker (see Fig. S4). The gene disruption construct was introduced into a *Δmus51*::*phleoR* strain impaired for NHEJ (50). Two independent hygromycin B-resistant (hygR) transformants were obtained. In order to purify *ΔPa_6_1340*::*hygR* from the *Δmus51*::*phleoR* mutation, we crossed both primary transformants with the *S* strain. Interestingly, we observed partially demelanized ascospores in the progeny of both crosses. Furthermore, when homokaryotic ascospores were sown on G+YE germination medium, only half of the progeny germinated, and those were only (hygS) melanized ascospores. This suggested that the (hygR) *ΔPa_6_1340*::*hygR* ascospores were the partially demelanized ones and that they were not able to germinate. These crosses were repeated on M2 medium supplemented with tricyclazole, a fungicide impairing melanin synthesis and provoking spontaneous germination of ascospores in *P. anserina* (23). We collected ascospores directly projected on M2 medium supplemented with hygromycin B, and we isolated (hygR) germinating thalli. These thalli were fragmented and homokaryotic (hygR, phleoS) *ΔPa_6_1340*::*hygR* strains of each mating type were purified (see Materials and Methods). Deletion of *Pa_6_1340* CDS (*Δgun1*) in these strains was verified by Southern blot analysis (see Fig. S4), and mutant phenotypes were characterized. In homozygous *ΔPa_6_1340*::*hygR* × *ΔPa_6_1340*::*hygR* crosses, ascospores exhibited demelanization and completely lost their ability to germinate on G+YE medium, a phenotype opposite to that of the *gun1^SG^* mutant (*Δgun1* in Fig. 1). Genetic analyses of heterokaryotic ascospores showed that this phenotype due to *ΔPa_6_1340*::*hygR* deletion was recessive and that *ΔPa_6_1340*::*hygR* segregated with a second division segregation (SDS) rate of 55% (a detailed genetic analysis is provided in Materials and Methods under “tetrad analysis in the *gun1^SG^* and in the *Δgun1* strains”). Fertility in the *ΔPa_6_1340*::*hygR* strain was affected too: perithecium production in a *ΔPa_6_1340*::*hygR* × *ΔPa_6_1340*::*hygR* (*Δgun1*) cross was slightly reduced, and ascospore production was significantly diminished compared to a wild-type cross (see Fig. S2). It has been shown that the formation of the appressorium in M. oryzae and the germination of melanized ascospores in *P. anserina* are two processes sharing common regulatory elements (16). We found that in the *ΔPa_6_1340*::*hygR* mutant, breaching of cellophane (a process involving appressorium development in *P. anserina*) was delayed compared to the wild type (Table 1). However, microscopic observations did not detect any morphological defect of appressorium development (Fig. S3). Importantly, introduction of the wild-type allele of *Pa_6_1340* carried on the *pGUN1* plasmid (see Materials and Methods) into the *ΔPa_6_1340*::*hygR* genome restored wild-type phenotypes, thus showing that the deletion of *Pa_6_1340* (*Δgun1*) was responsible for the mutant phenotypes (Fig. 1, 2, and 4; see also Fig. S2).

Finally, we addressed the question of whether the phenotypes in the *gun1^SG^* mutants were due to the I441N mutation in *Pa_6_1340* or not, by testing whether *ΔPa_6_1340*::*hygR* and *gun1^SG^* are alleles of the same gene by means of a complementation test. First, we genetically determined that spontaneous germination in *gun1^SG^* was a recessive trait, a prerequisite for the complementation test. These genetic analyses also showed that *gun1^SG^* segregated with a SDS rate of 54%, very similar to that of *ΔPa_6_1340*::*hygR* (55%), suggesting that *ΔPa_6_1340*::*hygR* and *gun1^SG^* mutants could be allelic (see “Tetrad analysis in the *gun1^SG^* and in the *Δgun1* strains” in Materials and Methods). We then crossed *gun1^SG^* with *ΔPa_6_1340*::*hygR* strains, reasoning that if *ΔPa_6_1340*::*hygR* and *gun1^SG^* strains are allelic, the SDS asci of the F_1_ progeny will be composed of four *ΔPa_6_1340*::*hygR/gun1^SG^* heterokaryotic ascospores showing no functional complementation: these ascospores can germinate spontaneously on M2 medium supplemented with hygromycin B (note that, since the *gun1^SG^* phenotype is not fully penetrant, the number of ascospores germinating per ascus can vary); in contrast, if *gun1^SG^* and *ΔPa_6_1340*::*hygR* strains are not allelic (i.e., they are alleles belonging to two different loci: the “*Pa_6_1340*” locus and the “*GUN1*” locus), functional complementation leading to restoration of wild-type phenotype (no spontaneous germination) is expected in SDS asci. In the F_1_ progeny of the *gun1^SG^* × *ΔPa_6_1340*::*hygR* cross, we observed in SDS asci that up to four heterokaryotic ascospores germinated spontaneously on M2 medium supplemented with hygromycin B (no functional complementation), showing that *gun1^SG^* and *ΔPa_6_1340* strains were allelic. This demonstrated that *Pa_6_1340* was the gene mutated in the *gun1^SG^* mutant responsible for the spontaneous germination phenotype. We therefore named *Pa_6_1340*, *GUN1*. This evidence was confirmed when we showed that the *gun1^SG^* mutant was also complemented by ectopic integration of a wild-type copy of *Pa_6_1340*/*GUN1* carried by the pGUN1 plasmid (Table 2; see also Materials and Methods).

**TABLE 2:** Functional complementation of *gun1^SG^* by pGUN1: *gun1^SG^* × *gun1^SG^ pGUN1* (*pBC-GUN1-genR*)^*a*^

Medium and marker	No. of germinated spores
M2	
genR	0
genS	11
Total	42
G+YE	
genR	18
genS	13
Total	42

a Forty-two homokaryotic ascospores were sown on both M2 and G+YE media.

### *P. anserina* possesses two CATs.

A BLASTP search on the *P. anserina* predicted CDS database (http://podospora.i2bc.paris-saclay.fr/) identified a second CAT encoded by the *Pa_3_7660* putative CDS. Compared to GUN1, this putative enzyme did not harbor any localization signal. Similarly to other *Fungi*, *P. anserina* may be endowed with two types of CAT, one located in peroxisomes and in mitochondria (GUN1) and one remaining in the cytoplasm (Pa_3_7660). To confirm this hypothesis, we undertook a phylogenetic analysis of CATs in *Fungi* and searched the homologs of *GUN1* in the genome sequence of representative fungal species (see Materials and Methods). In these species, two CATs were always identified, except for S. cerevisiae in which three CATs were identified as previously shown (49). The protein sequences were aligned, and the corresponding phylogenetic tree was built (see Fig. S5). The phylogeny of CATs in *Fungi* clearly indicated that there are two main types of CATs in *Fungi*: the putative peroxisomal/mitochondrial CAT, including GUN1, A. nidulans AcuJ, and M. oryzae Pth2/Crat1, and the putative “cytoplasmic” CATs, including *P. anserina* Pa_3_7760, A. nidulans FacC, and M. oryzae Crat2. Careful analysis of protein sequences indicated that proteins of the former type all contained the appropriate sequence signals to locate in peroxisomes and in mitochondria. In addition, search for orthologs through OrthoDB (51) did not identify orthologs for either *GUN1* or *Pa_3_7760* in plants or in bacteria.

*Pa_3_7760* putative CDS was renamed *GUP1* for *GUN1 Paralog 1*. To determine the function of this second CAT and assess its role in ascospore germination, we undertook a targeted gene disruption of *GUP1*. The *Pa_3_7660* CDS was replaced by a phleomycin resistance marker through homologous recombination in a *Δmus51*::*genR* strain (see Materials and Methods and Fig. S6). The *Δgup1* strain was purified from the *Δmus51* mutation by crossing primary transformants with the wild-type *S* strain followed by the selection of (phleoR genS) homokaryotic ascospores of the *Δgup1* genotype in the progeny. *Δgup1* ascospores had no melanization defect and germinated on G+YE medium as the wild type. Importantly, when *Δgup1* homokaryotic ascospores were sown on M2 medium, no spontaneous germination was observed, leading us to conclude that *GUP1* deletion had no effect on ascospore melanization and germination in *P. anserina*. In addition, the *Δgup1* strain differentiated wild-type mycelium and exhibited wild-type fertility, ascospore production, appressorium development, and cellophane breaching (Fig. 2 and Table 1; see also Fig. S2 and S3). We then constructed the *Δgun1 Δgup1* double mutant (see Materials and Methods) and observed that *Δgun1 Δgup1* homokaryotic ascospores exhibited impaired melanization and lack of germination similarly to the *Δgun1* ascospores.

### GUN1 and GUP1 functions are conserved in *Fungi*.

Previous studies in *Fungi* have demonstrated that CATs play an important role in primary metabolism and carbon source utilization. In particular, it has been shown that knockout strains of the cytoplasmic CAT do not grow on acetate, whereas mutant strains of the peroxisomal/mitochondrial CAT do not grow on acetate and on media containing fatty acids, such as oleic acid (36, 39, 47–49). To assess the role of GUN1 and GUP1 in carbon source utilization, we monitored the growth of the different mutant strains on acetate and oleic acid. As shown in Fig. 2, the wild-type *S* strain was able to grow on all the tested media, including the Tween 40 control (Tween 40 is necessary to solubilize oleic acid), indicating that *P. anserina* was able to use this detergent as a carbon source. Remarkably, the *gun1^SG^* strain, as well as the *gun1^SG^ pGUN1* complemented strain, grew as the wild type. The *Δgun1*, *Δgup1*, and the double *Δgun1 Δgup1* mutants grew as the wild type on dextrin (M2 medium), and the *Δgun1* and *Δgun1 Δgup1* strains exhibited slightly reduced growth on Tween 40, almost no growth on oleic acid, and no growth on acetate, whereas *Δgup1* growth was impaired only on acetate. The *Δgup1 pGUP1* strain had restored wild-type growth on acetate, indicating that wild-type *GUP1* complemented *Δgup1* deletion. This confirmed that *GUP1* function was required in *P. anserina* on acetate. Similarly, wild-type growth was restored on Tween 40 and oleic acid in both *Δgun1 pGUN1* and *Δgun1 Δgup1 pGUN1* complemented strains, indicating that *GUN1* function was required for Tween 40 and oleic acid utilization. Strikingly, both the *Δgun1* and the *Δgun1 Δgup1* strains could grow on Tween 40 but not on oleic acid medium, although the latter contained the same amount of Tween 40 (0.5%; see Materials and Methods). This suggested that impaired growth on oleic acid for *Δgun1* and *Δgun1 Δgup1* strains was due to a toxic effect of oleic acid in these mutant strains. Overall, these data showed that the roles of both main types of CATs were conserved in *P. anserina*: CATs of the AcuJ/Pth2/Crat1/GUN1 type are required for growth on acetate, as well as on long-chain fatty acids, whereas CATs of the FacC/Crat2/GUP1 type are required for growth on acetate.

### Structure prediction analysis of *gun1^SG^* loss of function.

MAFFT alignment with the protein sequences encoded by the fungal orthologs of *GUN1*, including the ortholog from Homo sapiens, revealed that the isoleucine 441 mutated in the *gun1^SG^* mutant (I441N) was highly conserved in *Fungi*: it is conserved in *Pezizomycotina*, *Saccharomycotina*, *Mucoromycota*, and *Basidiomycota* (see Fig. S7). Using I-TASSER, we carried out three-dimensional (3D) structure prediction of GUN1, and we compared this to the 3D structure of the murine CAT (PDB 2H3P, 34% identity). As can be seen in Fig. 3C, the overall structure of both enzymes was well conserved, showing that the 3D modeling of GUN1 was congruent. Based on this GUN1 3D model, we could localize the isoleucine 441 near the extremity of an α-helix. Since the hydrophobicity of amino acids is paramount in α-helix formation, we wondered whether the substitution of aliphatic isoleucine 441 by polar asparagine could destabilize the α-helix and/or the whole protein. We determined the Gibbs free-energy Gap (ΔΔ*G*) induced by the I441N substitution in *gun1^SG^* with STRUM (52). The calculated ΔΔ*G* of 2.11 kcal mol^−1^ was indeed indicative of a destabilization of the *gun1^SG^* mutant protein, but this low ΔΔ*G* value (<6 kcal mol^−1^) was indicative of a local destabilization of the protein rather than a complete destabilization (53). In line with this, we did not notice any stability issues in both chimera reporters gun1^SG^-mCherry and gun1^SG^-mCherry-AKI compared to GUN1-mCherry and GUN1-mCherry-AKI, respectively, in our microscopic observations (see below). We also used the PROVEAN (Protein Variation Effect Analyzer) analysis tool to determine the impact of the I441N substitution on GUN1 function. PROVEAN is a software tool predicting the potential deleterious effect of a point mutation (usually amino acid substitutions or indel). In keeping with the recessive nature of the *gun1^SG^* mutation, the calculated PROVEAN score of −6.63, far below the predefined cutoff of −2.5, was predictive of a “deleterious” loss-of-function effect of the I441N substitution on GUN1 function (54, 55).

### CAT activity decreases in *gun1^SG^* and *Δgun1* strains.

We measured the CAT activity in different mutant strains in protein extracts from mycelium grown on M2 medium (Fig. 4). It is worth mentioning that we failed to measure CAT activity in ascospores, and we could only obtain reliable results in mycelia (see Materials and Methods). Compared to the wild type, the CAT activity in the mycelium was greatly reduced in *Δgun1*, *Δgun1 Δgup1*, and *gun1^SG^* mutants. Significantly, CAT activity was restored to the wild-type level in the *Δgun1 pGUN1* and *Δgun1 Δgup1 pGUN1* complemented strains, confirming that lack of *GUN1* was responsible for reduced CAT activity. In the complemented *gun1^SG^ pGUN1* strain, the CAT activity was even higher than in the wild-type pointing to a role of CAT activity increase in the restoration of the wild-type phenotype in *gun1^SG^ pGUN1*. Interestingly, CAT activity in the *Δgup1* strain was similar to wild-type CAT activity, a result in line with previous observations in M. oryzae showing that CAT activity in *Δcrat2* (the ortholog of *GUP1*) mutants was not altered (37). These results, showing a decreased CAT activity in *gun1^SG^*, and hence a loss of function of *gun1^SG^*, were congruent with the modelized “deleterious” effect of the I441N mutation on *gun1^SG^* function and the recessivity of the *gun1^SG^* phenotype. However, the fact that similar reduced CAT activity was measured in *gun1^SG^* and *Δgun1* (*P* < 0.05) strains suggested that the CAT activity measured in the mycelium did not account for the difference in phenotype between *gun1^SG^* and *Δgun1* mutants (i.e., germination and melanization of ascospores, acetate, and acid oleic growth).

### Subcellular localization of GUN1 and gun1^SG^ proteins.

**FIG 5:**
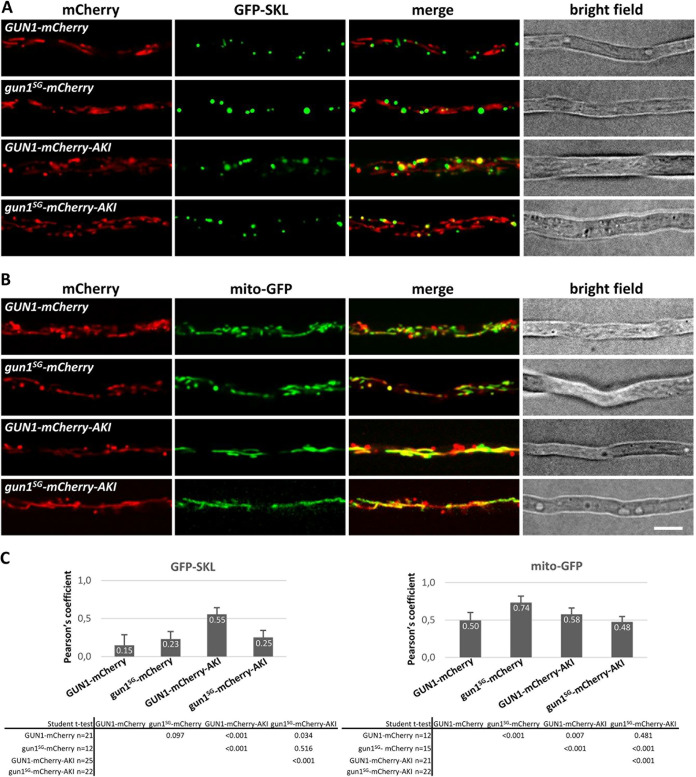
Spinning disk imaging of GUN1 and GUN^SG^ subcellular localization in mycelium. GUN1 and gun1^SG^ have been tagged with the mCherry or the mCherry-AKI (bearing GUN1 PTS1 AKI triad) fused in the C terminus. (A) Every strain analyzed carries the GFP-SKL marker tagging the peroxisomes. (B) Every strain carries the mito-GFP reporter tagging mitochondria. Scale bar, 5 μm. (C) Colocalization analysis data. The Pearson’s correlation coefficient (PCC) was calculated for each genotype and is indicated in the diagram. The *P* values of the *t* tests determined between the genotypes analyzed are given in the tables below each diagram. The numbers of analyzed ROIs per genotype are indicated in the tables.

**FIG 6:**
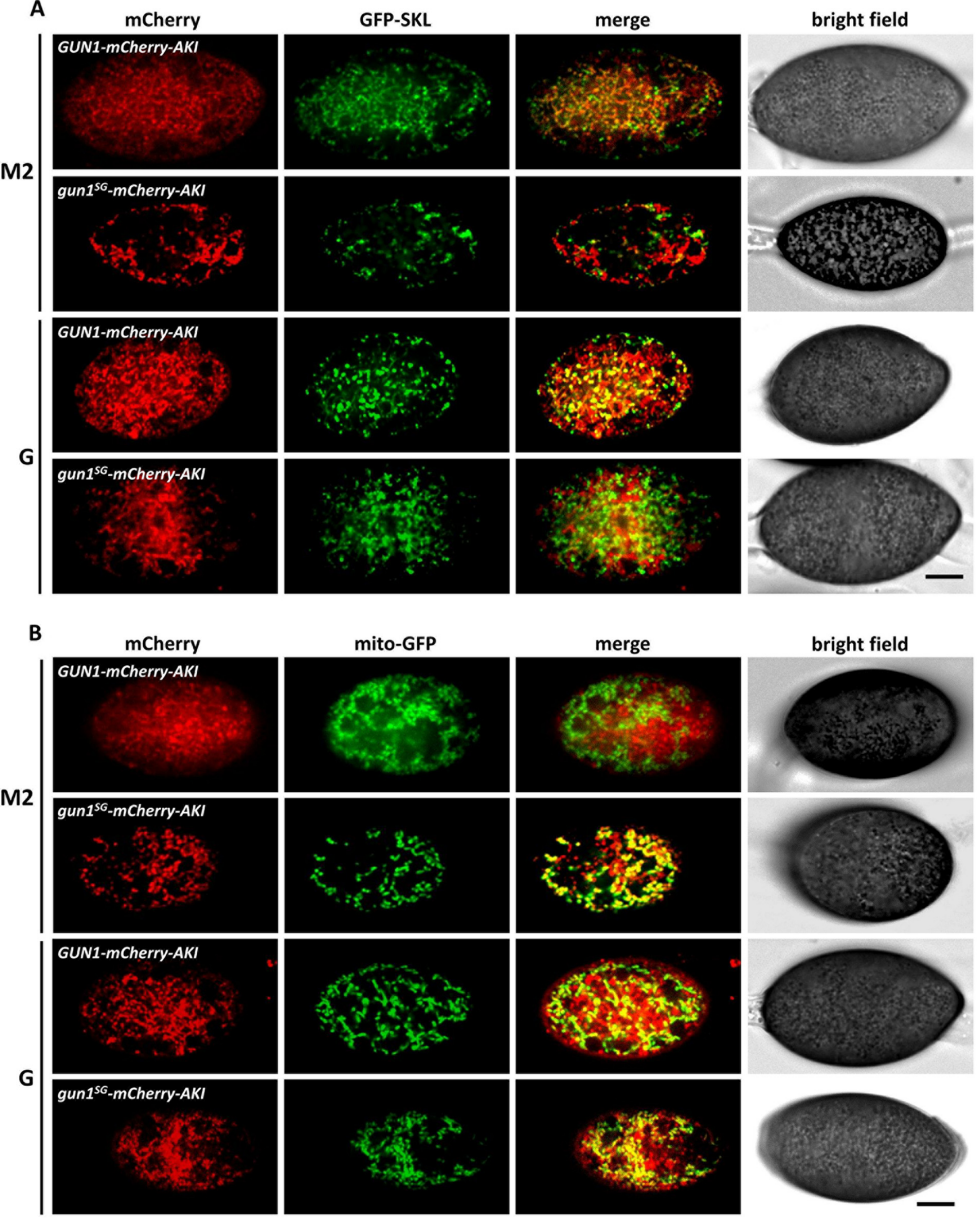

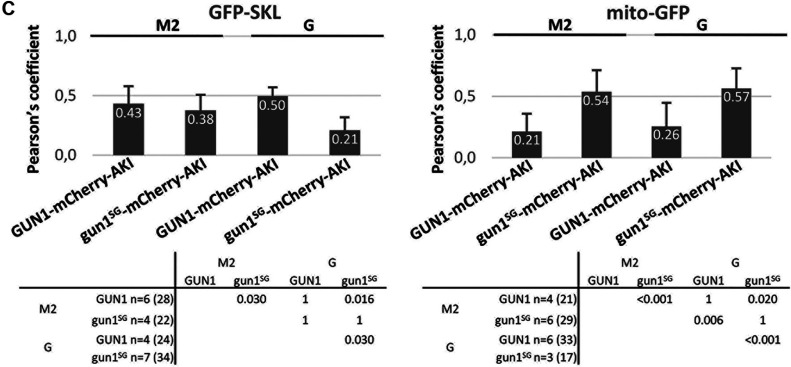
Spinning disc imaging of GUN1-mCherry-AKI and gun1^SG^-mCherry-AKI in ascospores. All the ascospores imaged carry the *Papks1^136^* mutation (genotype *Papks1^136^/Papks1^136^*) partially impairing ascospore melanization and making ascospores transparent for fluorescence imaging. (A) Every strain carries the GFP-SKL reporter tagging peroxisomes. (B) Every strain carries the mito-GFP reporter tagging mitochondria. M2 liquid medium, no induction of ascospore germination; G liquid medium, ascospore germination induction. Scale bar, 5 μm. (C) Colocalization analysis data. The Pearson’s correlation coefficient (PCC) was calculated for each genotype under the different incubation conditions (M2 and G) and is indicated in the diagram. *Post hoc* pairwise comparisons were performed using a Bonferroni-corrected *t* test with the Satterwaithe approximation (unequal variances), considering a family-wise error rate of 5%. *P* values of each comparison are reported in the tables below each diagram. The number of analyzed ascospores (*n*) and the number of analyzed ROIs (in parentheses) are indicated in the tables.

Previous studies carried out on GUN1-type CATs in other fungal species have indicated that these enzymes could be localized in peroxisomes and in mitochondria (39, 56). Analysis of GUN1 protein sequence using wolfPSORT showed that GUN1 is probably located in both peroxisomes and mitochondria. Accordingly, scanning GUN1 protein sequence with MitoFates allowed us to identify a mitochondrial targeting sequence (MTS), and we manually identified the “AKI” tripeptide at the C-terminal end of the protein sequence as a type 1 peroxisomal targeting sequence (PTS1) (57) (Fig. 3). In order to investigate GUN1 subcellular localization, as well as the impact of the *gun1^SG^* mutation on its subcellular localization, we tagged both GUN1 and gun1^SG^ proteins with mCherry and with mCherry-AKI, a modified mCherry version bearing the putative PTS1 peroxisome targeting signal of GUN1 in the C terminus (Fig. 3). As previously mentioned, *GUN1* is specifically induced in ascospores during germination (Demoor, unpublished). To ensure that expression of the fusion proteins was under the control of the native *GUN1* regulatory sequences, we tagged the endogenous *GUN1* alleles (at the *GUN1 locus*) through insertion of the mCherry and mCherry-AKI coding sequences in 3′ of *GUN1* and *gun1^SG^* CDS by homologous recombination (see Fig. S8 and Materials and Methods). Four tagged strains were obtained: *GUN1-mCherry*, *gun1^SG^-mCherry*, *GUN1-mCherry-AKI*, and *gun1^SG^-mCherry-AKI*. Importantly, *GUN1-mCherry* and *GUN1-mCherry-AKI* tagged strains germinated like the wild type, whereas *gun1^SG^-mCherry* and *gun1^SG^-mCherry-AKI* tagged strains germinated spontaneously on M2 medium like the *gun1^SG^* mutant. This suggested that the tagging by mCherry and mCherry-AKI did not modify GUN1 and gun1^SG^ functions during ascospore germination. Each strain was crossed with strains expressing green fluorescent protein (GFP) markers tagging either mitochondria (mito-GFP) or peroxisomes (GFP-SKL) to obtain double-tagged strains in the progeny (58, 59). Importantly, the presence of the *mito-GFP* or the *GFP-SKL* reporter genes did not modify ascospore germination. These double tagged strains allowed us to observe subcellular localization of GUN1 and gun1^SG^ in mycelium (Fig. 5) but not in melanized ascospores (data not shown). To that end, each strain was crossed with the *Papks1^136^* mutant producing partially demelanized ascospores in order to obtain all the double-tagged strains in a *Papks1^136^* genetic background in the progeny. In contrast to the *Papks1^193^* mutation, which leads to spontaneous ascospore germination (Fig. 1B), the *Papks1^136^* mutation did not modify ascospore germination. All the *Papks1^136^* double-tagged strains produced partially demelanized ascospores, allowing fluorescence microscopic observations within ascospores, and were isolated in both mating types (*mat*+ and *mat*–) in order to proceed to homozygous crosses for ascospore production and observation. Ascospores were observed either in M2 liquid medium (noninduction condition) or in G liquid medium for the induction of ascospore germination (Fig. 6). We observed in ascospores and in mycelium that GUN1-mCherry-AKI and gun1^SG^-mCherry-AKI could colocalize with both mito-GFP and GFP-SKL, showing that both GUN1- and gun1^SG^-mCherry-AKI reporter proteins could be found in mitochondria and in peroxisomes (Fig. 5). Colocalization of GUN1 and gun1^SG^-tagged proteins with mitochondria and peroxisomes was qualitatively determined by visual analysis of images and quantified by calculating the Pearson’s correlation coefficient (PCC) between the green and the red fluorescence signals. PCCs range from 1 for two images whose fluorescence signals are perfectly linearly related to −1 for two images whose fluorescence signals are perfectly, but inversely, related to one another, with intermediate values indicating partial colocalization. Values near zero reflect distributions of signals that are uncorrelated with one another (60). Careful comparison of the GFP-tagging pattern and the mCherry tagging pattern indicated that GUN1-mCherry-AKI and gun1^SG^-mCherry-AKI could be absent in some mitochondria or in some peroxisomes. This partial colocalization of both tagged proteins with peroxisomes and mitochondria was quantified by PCCs comprised between 0.21 (GUN1-mCHerry-AKI with mito-GFP in noninduced ascospores and gun1^SG^-mCherry-AKI with GFP-SKL in induced ascospores) and 0.58 (GUN1-mCherry-AKI with mito-GFP in the mycelium) (Fig. 5C and 6C). It should be stressed that the low PCC of 0.21 calculated for GUN1-mCHerry-AKI with mito-GFP in noninduced ascospores corroborated their evident lack of colocalization observed in Fig. 6B. In line with this, comparably low PCCs (0.15 and 0.23, respectively) were calculated for GUN1-mCherry and gun1^SG^-mCherry with GFP-SKL in the mycelium (Fig. 5C). Accordingly, Fig. 5A clearly shows that GUN1-mCherry and gun1^SG^-mCherry did not seem to colocalize with GFP-SKL. Compared to GUN1-mCherry-AKI, gun1^SG^-mCherry-AKI was the protein colocalizing the less with GFP-SKL in mycelium and in ascospores. Inversely, gun1^SG^-mCherry-AKI colocalization with mito-GFP in spontaneously germinating ascospores (*gun1^SG^-mCherry-AKI* ascospores germinate in M2 and G media) was more important (Fig. 6B and C). In comparison, GUN1-mCherry-AKI colocalization with mito-GFP was significantly lower in ascospores under induced (PCC = 0.26) and noninduced (PCC = 0.21) conditions for germination (Fig. 6B and C). Altogether, these data showed that the distribution of gun1^SG^-mCherry-AKI in mitochondria and in peroxisomes was different from the one of GUN1-mCherry-AKI, especially in ascospores where gun1^SG^-mCherry-AKI localized significantly more in mitochondria and less in peroxisomes than GUN1-mCherry-AKI.

We observed that GUN1-mCherry and gun1^SG^-mCherry colocalized only with the mito-GFP reporter in ascospores (see Fig. S9), as well as in mycelium (Fig. 5B). Similarly to the mycelium, a clear discrepancy in the localization of both GUN1- and gun1^SG^-mCherry-tagged proteins and the GFP-SKL marker was observed in ascospores (see Fig. S9A), and low PCCs (from 0.01 to 0.06) were measured. This observation was consistent with previous studies on the localization of *G. zeae* CAT1-GFP (the ortholog of GUN1) showing that adding the GFP in C terminus of the protein masked the PTS1 signal of CAT1 (48). More generally, it has been shown that adding a tag after a C-terminal PTS1 signal abolishes import into peroxisomes, suggesting that GUN1-mCherry and gun1^SG^-mCherry could be mislocalized (61, 62). It is worth noting that ascospores in the *GUN1-mCherry* tagged strain germinated as wild type and ascospores in *gun1^SG^-mCherry* tagged strain germinated spontaneously, strongly suggesting that mislocalization of GUN1- or of gun1^SG^-mCherry did not affect ascospore germination. In contrast, whereas *GUN1-mCherry-AKI* and *gun1^SG^-mCherry-AKI* strains grew as the wild type on the different media tested, the *GUN1-mCherry* and *gun1^SG^-mCherry* strains did not grow on oleic acid (see Table S3). This result suggested that peroxisomal localization of GUN1 (and gun1^SG^) was required for oleic acid metabolism.

### Cross-talk between GUN1 and the MAPK PaMpk2 pathway upstream of the PaNox2/PaPls1 complex.

**FIG 7:**
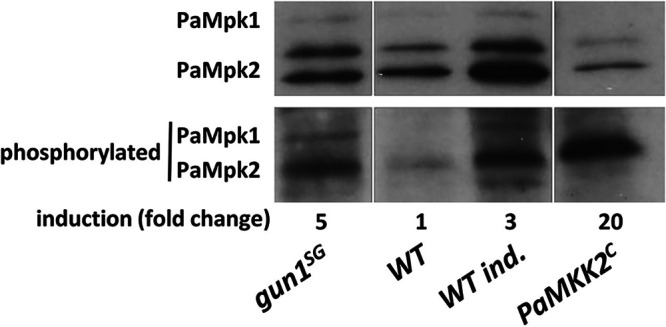
Western blot analysis of PaMpk2 phosphorylation in ascospores. Proteins were extracted from genetically homogeneous ascospores. Anti-p44 and anti-phospho-p44 antibodies recognizing both PaMpk1/PaMpk2 and p-PaMpk1/p-PaMpk2, respectively, were used. For ascospore germination induction (WT ind.), ascospores were placed on G+YE medium for 2 h before protein extraction (see Materials and Methods). *PaMKK2^c^* constitutive mutation induces PaMpk2 phosphorylation and spontaneous ascospore germination. The original blots were spliced, and noncontiguous lanes are separated.

**FIG 8:**
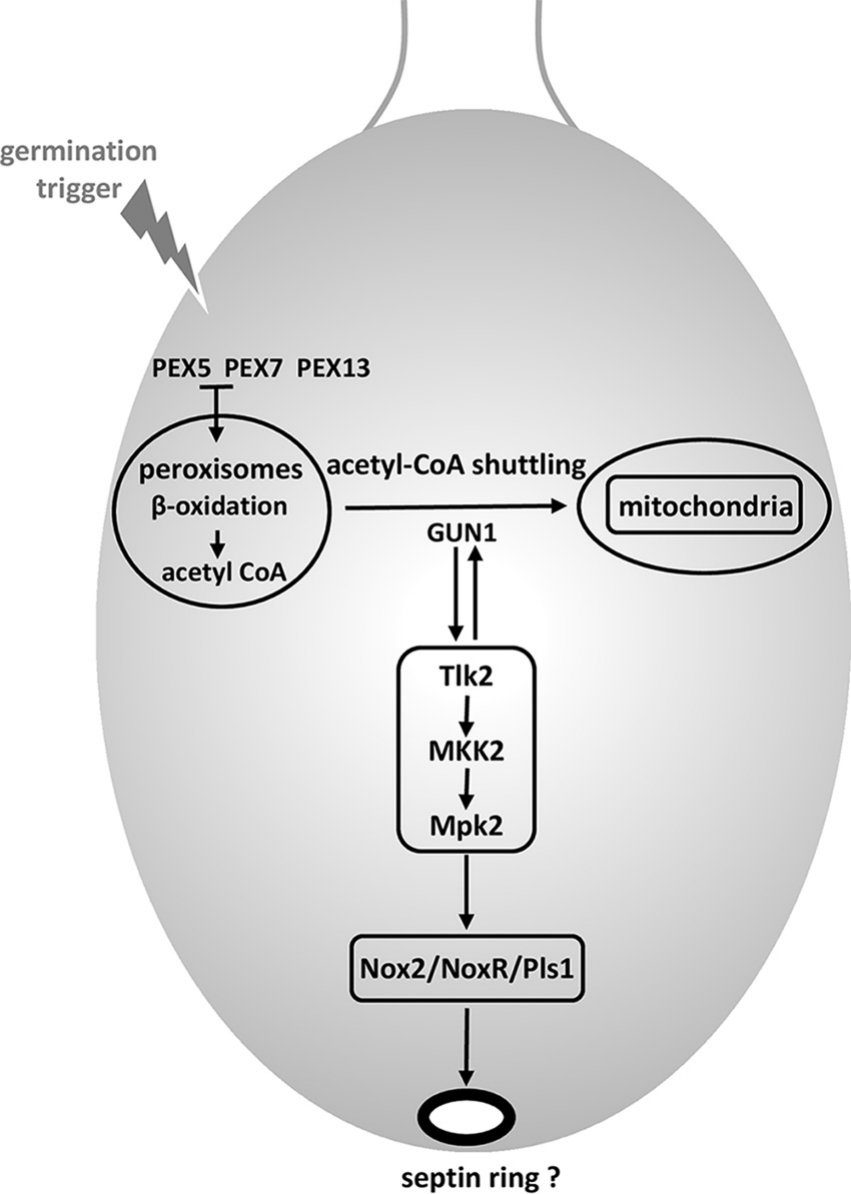
Regulation of ascospore germination. Breaking of dormancy is initiated by germination triggers: ammonium acetate and Bacto peptone *in vitro*. The activities of the PTS1 and PTS2 import receptors PEX5 and PEX7, as well as that of the importomer component Pex13, are required for germination. We speculate that GUN1-driven acetyl-CoA shuttling to mitochondria activates the Mpk2/FUS3 MAPK pathway, which in turn activates the NADPH oxidase complex Nox2/Pls1/NoxR. Reciprocally, the Mpk2/FUS3 MAPK module requires GUN1 function to activate ascospore germination. Whether activation of the Nox2 complex sets up a septin ring and actin cytoskeleton rearrangement at the germination pore in a similar manner as in M. oryzae appressorium remains to be addressed.

We have shown in previous studies that the MAPK *PaMpk2* pathway, *PaNox2*, its regulatory subunit *PaNoxR*, and *PaPls1* are essential for ascospore germination in *P. anserina*: the deletion of these genes blocks ascospore germination; inversely, constitutive phosphorylation/activation of PaMpk2 in the *PaMKK2^c^* mutant carrying a constitutively active allele of *PaMKK2*, triggers spontaneous ascospore germination (Fig. 7) (8–10, 17). We took advantage of the spontaneous germination phenotype of the *gun1^SG^* mutant to conduct epistasis studies in order to place *GUN1* in the regulatory cascade triggering ascospore germination. These studies were carried out by crossing *gun1^SG^* with the *ΔPaMpk2*, *ΔPaPls1*, and *ΔPaNox2* strains. A total of 48 homokaryotic spores were sown on M2 medium for each cross, but none of the germinated spores presented the *ΔPaMpk2*, *ΔPaPls1*, or *ΔPaNox2* deletions (Table 3). This clearly showed that PaMpk2, PaPls1, and PaNox2 were required for germination in *gun1^SG^* ascospores. In keeping with this, we found that PaMpk2 was constitutively phosphorylated in *gun1^SG^* ascospores. In absence of germination induction, PaMpk2 was phosphorylated at an even higher level in *gun1^SG^* ascospores than in wild-type ascospores induced for germination (Fig. 7). We then crossed the *Δgun1* strain with the *PaMKK2^c^* mutant showing spontaneous ascospore germination (17). In the progeny of this cross, 305 homokaryotic ascospores were sown on M2 medium and 208 on G + YE medium (Table 4). Strikingly, no (hygR) *Δgun1* bearing ascospore germinated on either medium. In addition to the very low germination rate observed (see below), the very low number of wild-type ascospores germinating on G + YE medium suggested that genetic linkage decreased recombination between the *PaMKK2^c^* transgene insertion site and the *GUN1* locus, leading to a reduction in the expected number of Δ*gun1 PaMKK2^c^ ascospores in the* progeny. To test this hypothesis independently of the germination defect due to Δ*gun1* deletion, we crossed the *PaMKK2^c^* strain with the *GUN1-mCherry-AKI* strain obtained by homologous recombination at the *GUN1* locus (see Materials and Methods, Fig. S8). 84/123 homokaryotic ascospores germinated on G + YE medium (Table S4). In this cross, we observed a reduced number of progeny with recombined genotype (χ^2^-test, *P* value = 1.5 × 10^−6^) arguing for genetic linkage between the *PaMKK2^c^* transgene insertion site and the *GUN1* locus (recombination frequency, *r* = 0.202; genetic distance = 20.2 cM). This recombination frequency (*r* = 0.202) was used in the analysis of the Δ*gun1* × *PaMKK2^c^* cross to calculate the “expected” size of each genotypic category in the progeny (Table 4). In particular, 21 Δ*gun1 PaMKK2^c^* ascospores were expected on G + YE and 30.5 on M2 (Table 4). We also determined that the germination rate of *PaMKK2^c^* ascospores was 49% (41/83) on G + YE and 22% (27/122) on M2. Because spontaneous ascospore germination on M2 due to *PaMKK2^c^* was 22% in this cross, we estimated that if Δ*gun1* had no influence on germination, a maximum of 6.8 ascospores of the Δ*gun1 PaMKK2^c^* genotype might have germinated. Finally, the lack of (hygR) ascospores in the progeny showed that Δ*gun1* impaired both ascospore germination on G + YE as well as *PaMKK2^c^*-induced spontaneous ascospore germination on M2. Altogether, these data clearly indicated that *gun1^SG^* triggered spontaneous ascospore germination through the activation of the PaMpk2 MAPK pathway and they also showed that GUN1 function was required when spontaneous ascospore germination was triggered by the constitutive activation of PaMpk2 (Fig. 8).

**TABLE 3:** Epistasis studies between *gun1^SG^* and *ΔPaPls1*, *ΔPaNox2*, and *ΔPaMpk2* strains

Strain and marker	No. of germinated spores
*gun1^SG^* mutant × *ΔPaPls1*::*hygR* mutant^*a*^	
hygR	0
hygS	20
Total	48
*gun1^SG^* mutant × *ΔPaNox2*::*phleoR* mutant^*a*^	
phleoR	0
phleoS	13
Total	48
*gun1^SG^* mutant × *ΔPaMpk2*::*hygR* mutant^*a*^	
hygR	0
hygS	26
Total	48

a Forty-eight homokaryotic ascospores were sown on M2 medium.

**TABLE 4:** Epistasis study between *PaMKK2^C^* and *Δgun1*: *PaMKK2^C^-phleoR* X *Δgun1::hygR*^*a*^

		M2		G + YE		
Phenotype	Genotype	Germinated	Expected	Germinated	Expected	
[hygR, phleoS]	*Δgun1*	0	122	0	83	(1 − *r*)/2
[hygS, phleoR]	*PaMKK2^C^*	27 (22%)^*b*^	122	41 (49%)^*b*^	83	(1 − *r*)/2
[hygS, phleoS]	*+*	0	30.5	10 (48%)^*b*^	21	r/2
[hygR, phleoR]	*Δgun1 PaMKK2^C^*	0 (6.8)^*c*^	30.5	0 (10.4)^*c*^	21	r/2
		Total: 27	Sown: 305	Total: 51	Sown: 208	

a 305 homokaryotic ascospores were sown on M2 medium and 208 were sown on G + YE medium.

b (%), germination rate (germinated/expected): *PaMKK2^C^* on M2:27/122; on G + YE:41/83; *+: 10/21*. The number of “germinated” ascospores is indicated (Total). “Expected”: the putative size of the different genotypic categories was calculated using the recombination frequency (*r* = 0.202) between the *PaMKK2^C^* insertion site and the *GUN1* locus: for the parental genotypes: sown × (1 − *r*)/2; for the recombined genotypes: sown × *r*/2.

c (6.8) and (10.4): putative number of germinating ascospores calculated when taking into account both the recombination frequency and the germination rate of *PaMKK2^C^* spores on M2 or on G + YE medium: 6.8 = 305 × (*r*/2) × (27/122); 10.4 = 208 × (*r*/2) × (41/83).

### *gun1^SG^*-induced ascospore germination requires PEX5 and PEX13.

Peroxisomes are key organelles for germination. It has been shown that mutants of the peroxisomal matrix protein import machinery (such as *Δpex5*, *Δpex7*, and *Δpex13* mutants) produce partially demelanized ascospores impaired for germination (29, 33, 63). We crossed *gun1^SG^* with the *Δpex5 Δpex7* double mutant and with the *Δpex13* mutant and checked whether *Δpex5 gun1^SG^*, *Δpex7 gun1^SG^*, and *Δpex13 gun1^SG^* double mutants could germinate when sown on G+YE germination medium (Tables 5 and 6). None of the germinated ascospores carried *Δpex5 or Δpex13*, showing that *Δpex5*, *Δpex13*, *Δpex5 gun1^SG^*, and *Δpex13 gun1^SG^* ascospores did not germinate. The fact that *Δpex5* and *Δpex13* mutants were epistatic over the *gun1^SG^* strain indicated that the function of both genes was required for *gun1^SG^*-induced ascospore germination.

**TABLE 5:** Epistasis studies between *Δpex5*, *Δpex7*, and *gun1^SG^*: *Δpex5 Δpex7* (*Δpex5*::*hygR Δpex7*::*phleoR*) × *gun1^SG^* strains^*a*^

Strain (markers)	No. of germinated spores
*Δpex7 gun1^SG^* mutant (hygS, phleoR)	27*
*gun1^SG^* mutant (hygS, phleoS)	20*
*+* strain (hygS, phleoS)	7
*Δpex5 gun1^SG^* mutant (hygR, phleoS)	0
*Δpex5* mutant (hygR, phleoS)	0
*Δpex5 Δpex7* mutant (hygR, phleoR)	0
*Δpex7* mutant (hygS, phleoR)	2
*Δpex5 Δpex7 gun1^SG^* mutant (hygR, phleoR)	0
Total	152

a One hundred fifty-two homokaryotic ascospores were sown on G+YE medium. *, Ascospores of this genotype show spontaneous germination on standard M2 medium.

**TABLE 6:** Epistasis study between *Δpex13* and *gun1^SG^*:*Δpex13* (*Δpex13*::*hygR*) × *gun1^SG^* strains^*a*^

Strain (marker)	No. of germinated spores
*gun1^SG^* mutant (hygS)	14*
*+* strain (hygS)	5
*Δpex13* mutant (hygR)	0
*Δpex13 gun1^SG^* mutant (hygR)	0
Total	64

a Sixty-four homokaryotic ascospores were sown on G+YE medium. *, Ascospores of this genotype show spontaneous germination on standard M2 medium.

*PEX7* is located approximately 810,000 bp away from *GUN1* on chromosome 6. Therefore, genetic linkage between both loci should lead to a reduced number of recombinant *Δpex7 gun1^SG^* progeny compared to *Δpex7* parental progeny, regardless of any genetic interaction between *Δpex7* and *gun1^SG^* strains (i.e., suppression or epistasis). Interestingly, only 2 *Δpex7* ascospores germinated, a really low number compared to the 27 recombinant *Δpex7 gun1^SG^* ascospores that germinated. This result, opposite to what could be expected in case of genetic linkage only, suggested that *Δpex7* ascospores were strongly impaired for germination and that the *gun1^SG^* strain could suppress this defect in the recombinant *Δpex7 gun1^SG^* ascospores. However, it was previously shown that in a heterozygous cross, the germination of *Δpex7* ascospores is only moderately affected (29). Hence, the fact that only 2 *Δpex7* ascospores germinated could reveal complex genetic interactions between *Δpex7*, *Δpex5*, and *gun1^SG^* strains and, mostly, a non-cell-autonomous effect of the *Δpex5* mutation on the germination of ascospores as previously observed (29). We therefore directly tested the genetic interaction between *Δpex7* and *gun1^SG^* strains by crossing the *Δpex7* strain with the *gun1^SG^* strain (Table 7). Only 50/90 ascospores germinated on G + YE medium. We performed a χ^2^ test that determined that the different genotypic categories obtained were not statistically different in size (*P* = 0,059). In conclusion, no genetic interaction between *Δpex7* and *gun1^SG^* strains was observed, *gun1^SG^* had no suppressive effect on *Δpex7* ascospore germination defect. Accordingly, *Δpex7 gun1^SG^* ascospores had a similar melanization defect (gray color) as *Δpex7* ascospores, showing that *gun1^SG^* had no suppressive effect on the melanization defect of *Δpex7* ascospores.

**TABLE 7:** Epistasis study between *Δpex7* and *gun1^SG^*:*Δpex7* (*Δpex7*::*phleoR*) × *gun1^SG^* mutants^*a*^

Strain (marker)	No. of germinated spores
*gun1^SG^* mutant (phleoS)	13*
*+* strain (phleoS)	7
*Δpex7* mutant (phleoR)	20
*Δpex7 gun1^SG^* mutant (phleoR)	10*
Total	90

a Ninety homokaryotic ascospores were sown on G+YE medium. *, Ascospores of this genotype show spontaneous germination on standard M2 medium.

## DISCUSSION

Despite its importance in the fungal life cycle, the regulation of ascospore germination in filamentous fungi has not been thoroughly researched. In this study, we aimed at uncovering new actors of this regulation pathway in *P. anserina*. To that end, we conducted a direct genetic screen, a particularly powerful approach to identify new genes. A majority of the mutants isolated, including all suppressors of *ΔPaNox2* and *ΔPaPls1*, showed spontaneous but abnormal ascospore germination. However, for six mutants, germination proceeded as in the wild type through the germination pore at the tip of the ascospore. We hypothesized that in these mutants, ascospore dormancy was broken and that these were mutants specifically impaired in the control of ascospore germination. Here, we characterize the first of these six *Germination UNcontrolled-GUN* mutants (*gun1^SG^*). Although most of the spontaneous germination analyses were performed on M2 medium containing dextrin, the *gun1^SG^* mutant germinated on medium lacking carbon and nitrate sources, indicating that in this mutant, control of dormancy escaped possible nutrient stimuli (data not shown). Through whole-genome sequencing and genetic analyses, we showed that the gene mutated in *gun1^SG^* is the *Pa_6_1340* putative CDS. This CDS encodes a peroxisomal/mitochondrial carnitine-acetyltransferase (CAT), a key metabolic enzyme involved in acetyl-CoA shuttling between peroxisomes and mitochondria (45, 64). Gene expression of this CAT is significantly induced during ascospore germination, highlighting the pivotal role of this enzyme during this process (Demoor, unpublished). The mutation in *gun1^SG^* CDS is a substitution of the conserved isoleucine 441 by an asparagine (I441N). 3D modelization of GUN1 and of the mutated gun1^SG^ protein combined with *in silico* analyses of the stability of gun1^SG^ predict a moderate effect of the I441N substitution on the overall stability of the protein but a deleterious effect on its activity. This prediction correlates with the recessive nature of the *gun1^SG^* mutation, as well as the reduced CAT activity measured in *gun1^SG^*, both pointing to a loss of function of the *gun1^SG^* allele. Hence, GUN1 may act as an ascospore germination inhibitor. However, we also show that deletion of GUN1 leads to a complete lack of germination and a defect in melanin synthesis, indicating that GUN1 function is required for both processes and suggesting that the *gun1^SG^* allele is hypomorphic compared to *Δgun1*, which is a null allele. Impairment of both germination and melanization are frequently observed in mutants of peroxisomal import machinery, as well as in mutants of peroxisomal/mitochondrial β-oxidation (28, 58, 63). Indeed, in *P. anserina*, ascospores in the *ΔechA* mutant impaired for mitochondrial β-oxidation and in the *Δfox2* mutant impaired for peroxisomal β-oxidation show a reduced rate of germination (28). Interestingly, the *Δgun1* strain exhibits a complete lack of germination, suggesting that GUN1 and acetyl-CoA shuttling between peroxisomes and mitochondria is essential during activation of ascospore germination. As with many other fungi, we found a second CAT encoded in the *P. anserina* genome. Deletion of this second CAT encoding gene, *Pa_3_7660/GUP1* (*GUN1 Paralog 1*), does not show any ascospore germination defect nor melanization defect, suggesting that this CAT has no role in both processes. Furthermore, CAT activity is reduced in the *Δgun1* mutant and in the *gun1^SG^* strain but not in the *Δgup1* mutant (in mycelium). This finding is similar to what has been observed in M. oryzae where *Pth2* mutants (*GUN1* ortholog) show reduced CAT activity and reduced pathogenicity, while the *Δcrat2* knockout strain (*GUP1* ortholog) exhibits wild-type CAT activity and pathogenicity (37).

As previously found in *Fungi* (M. oryzae, A. nidulans, *G. zeae*, and S. cerevisiae), we show that in *P. anserina*, the peroxisomal/mitochondrial CAT GUN1 is required for growth on acetate and oleic acid while the cytosolic CAT GUP1 is required for growth on acetate only, showing strong conservation of the function of both enzymes in fungal primary metabolism (36, 39, 41, 47–49). Nonetheless, GUN1 is required for growth on fatty acids, but we also show that oleic acid is toxic for *Δgun1* mutants, as previously demonstrated for *pex2* mutants impaired for peroxisomal import (58). Indeed, both *Δgun1* and *Δgun1 Δgup1* strains grow better on Tween 40 control medium than on oleic acid medium containing similar amount of Tween 40. In contrast to peroxisomal import mutants such as *pex2* mutants, which are sterile in homozygous cross, fertility and ascospore production are only moderately decreased in *Δgun1* and *Δgun1 Δgup1* mutants.

To understand how the mutation in *gun1^SG^* triggers breaking of dormancy, we explored both *gun1^SG^* CAT activity and *gun1^SG^* subcellular localization. The *gun1^SG^* strain shows a loss in CAT activity in the mycelium similar to that of the *Δgun1* mutant. However, *Δgun1* and *gun1^SG^* mutant strains exhibit noticeable phenotype discrepancies: the *Δgun1* mutant produces nongerminating demelanized ascospores, while the *gun1^SG^* mutant produces spontaneously germinating melanized ascospores; also, the *gun1^SG^* mutant grows as the wild type on both oleic acid and acetate but not the *Δgun1* mutant. We were not able to measure CAT activity in ascospores and therefore to properly address the question of CAT activity during germination in *gun1^SG^* and *Δgun1.* The fact that both *Δgun1* and *gun1^SG^* mutants have a reduced CAT activity but different phenotypes is intriguing, and one cannot exclude that spontaneous germination in the *gun1^SG^* mutant could be due to an as-yet-unknown activity of GUN1, not related to acetyl-CoA shuttling but specifically involved in the control of dormancy in ascospores.

To explore how *gun1^SG^* causes spontaneous germination, both the wild-type GUN1 protein and the mutant *gun1^SG^* protein were tagged with mCherry or mCherry-AKI (AKI is the PTS1 peroxisomal import signal present in C terminus of GUN1), and colocalization studies with GFP-tagged peroxisomes and GFP-tagged mitochondria were performed. In the mycelium, as well as in ascospores, although GUN1 is found both in peroxisomes and in mitochondria, our data show that GUN1 is preferentially located in peroxisomes. The dual localization of GUN1 in mitochondria and in peroxisomes is in agreement (i) with the predicted localization of this CAT, bearing both a mitochondrial targeting sequence (MTS) at the N terminus and the peroxisomal targeting sequence (PTS1) AKI at the C terminus and (ii) with studies of GUN1 orthologs in other *Fungi* such as A. nidulans and *G. zeae* (39, 48). In contrast, M. oryzae Pth2 was shown to only localize in peroxisomes (36). Attention must be drawn to the fact that the main difference between GUN1 and gun1^SG^ is their respective distribution between mitochondria and peroxisomes, the gun1^SG^ mutant protein being predominantly present in mitochondria, while the GUN1 wild-type protein is preferentially located in peroxisomes. Furthermore, GUN1 seems to be almost exclusively located in peroxisomes in dormant ascospores not subjected to a germination trigger. These data suggest that the breaking of dormancy in wild-type and *gun1^SG^* ascospores may involve shuttling of gun1^SG^ from peroxisomes to mitochondria. We show that, in keeping with the role of mitochondria in ascospore germination, the mislocalization of GUN1-mCherry (and gun1^SG^-mCherry) solely in mitochondria does not impair the germination or melanization of ascospores. However, *GUN1-mCherry* and *gun1^SG^-mCherry* strains do not grow on oleic acid, in the same way as the *Δgun1* strain. A comparable effect has been reported in A. nidulans, where mislocalized AcuJ in the cytoplasm impairs growth on oleic acid but not on acetate (39). These data point to a central role of peroxisomal localization of GUN1 in oleic acid utilization independent of ascospore germination. However, it has been shown that peroxisomes are essential for germination in *P. anserina*, and our data confirm that *Δpex5* and *Δpex13* strains lacking PTS1-dependent peroxisomal import machinery cannot germinate (29, 33, 63). How ascospores carrying mislocalized GUN1 germinate is an open question. Given that GUN1 bears a PTS1-AKI at the C terminus (and no internal PTS2) and that the *Δpex5* mutant is epistatic over *gun1^SG^*, it is highly likely that GUN1 (and gun1^SG^) peroxisomal import is dependent on the PTS1 and not on the PTS2 import pathway. In this study, we could not determine any genetic interaction specifically between *Δpex7*, a deletion impairing PTS2-dependent import machinery, and the *gun1^SG^* mutation. However, we found that *Δpex7 gun1^SG^* ascospores germinated at a higher rate than *Δpex7* ascospores in the progeny when the *Δpex5 Δpex7* double mutant was crossed with the *gun1^SG^* strain. However, this result was specifically observed when the *Δpex5* mutation was present in the parental strain, confirming, as previously shown, that the *Δpex5* mutation has a non-cell-autonomous effect on ascospore germination (since it affects the germination of ascospores without the *Δpex5* deletion) (29). The *Δpex7* mutation has been shown to suppress some *Δpex5* mutant phenotypes during sexual reproduction (29); inversely, our results suggest that the *Δpex5* mutation could increase *Δpex7* germination defect and that *gun1^SG^* would suppress this *Δpex5-*dependent *Δpex7* germination defect. The complex genetic interactions taking place between these three genes suggest that they have intricate functions in peroxisomes and further studies will be required to clarify how the product of these genes interact in peroxisomes during ascospore germination.

Through the characterization of the *GUN* mutants, we sought to discover new genes that control dormancy in ascospores. Genetic approaches in *P. anserina* and, in particular, the amenability to perform epistasis studies makes this model fungus a powerful system for deciphering regulation pathways such as the one controlling ascospore germination. Here, we investigated the relationships between GUN1 and already-known actors of ascospore germination: the PaMpk2 MAPK pathway, the tetraspanin Pls1, and the NADPH oxidase Nox2 complex (4, 10, 17). We show that gun1^SG^ requires PaMpk2, PaNox2, and PaPls1 functions to induce the spontaneous germination of ascospores. Furthermore, we show that gun1^SG^ controls the PaMpk2 pathway by activating PaMpk2 phosphorylation. Altogether, these data demonstrate that on the one hand GUN1 acts upstream of the PaMpk2 pathway and the PaNox2/PaPls1 complex in the regulatory cascade controlling ascospore germination. But our data also show that on the other hand, *GUN1* function is required when spontaneous germination is triggered by the constitutive activation of the PaMpk2 pathway (Fig. 8).

Melanin is a major component of appressorium cell wall in M. oryzae where it is involved in turgor pressure generation (26). M. oryzae mutants, such as the *Pth2* mutant, showing impairment of melanin synthesis cannot build up the turgor pressure necessary in the appressorium for host penetration and are therefore nonpathogenic. As observed in the *Papks1^193^* mutant devoid of melanin biosynthesis, melanin in ascospores is important to avoid uncontrolled “accidental” germination (23). The melanization defect exhibited by the *Δgun1* ascospores is likely due to the lack of acetate supply to the dihydroxynaphthalene pathway involved in melanin biosynthesis, a defect shared by the *ΔechA* and *Δfox2 P. anserina* mutants, impaired in mitochondrial and in peroxisomal β-oxidation, respectively (23, 28). However, unlike *ΔechA* and *Δfox2* ascospores that germinate spontaneously, *Δgun1* ascospores do not, suggesting that the melanization defect in *Δgun1* ascospores is not sufficient to induce spontaneous germination. Accordingly, tricyclazole, a fungicide inhibiting melanin biosynthesis or the *Papks1^193^* mutation blocking melanin production (23), suppress the germination defect of *Δgun1* ascospores. Indeed, *Papks1^193^ Δgun1* ascospores germinate spontaneously (data not shown). Hence, it is likely that in *Δgun1* ascospores residual melanin in the cell wall is enough to avoid “accidental” spontaneous germination.

The fact that tricyclazole and *Papks1^193^* null mutation trigger germination of *Δgun1* ascospores suggests that these ascospores are competent for the formation of the germination peg and eventually hyphal growth but that they might be blocked in the formation of the germination pore. The formation of a pore in melanized ascospores is a process sharing similarities with the formation of the pore in M. oryzae appressorium (16). In this pathogenic fungus, the formation of the appressorial pore is preceded by the formation of a septin ring required for actin cytoskeleton remodeling and appressorial pore solidity. In-depth studies in M. oryzae have deciphered the intricate genetic signaling, culminating in the setting up of this septin ring (14, 65). Among other regulatory components, the FUS3/PMK1/PaMpk2 pathway and the NoxB (Nox2)/Pls1 complex play a key role in septin ring assembly. The fact that both pathways are also essential for ascospore germination in *P. anserina*, N. crassa, and *S. macrospora*, three species producing melanized ascospores, leads us to hypothesize that a similar process involving septin ring assembly and cytoskeleton remodeling may take place to initiate the formation of the germination pore. Given the similarities in the regulation of appressorium functioning and ascospore germination when those are melanized, we speculate that studying and discovering new genes controlling ascospore germination in *P. anserina* may lead to the discovery of new pathogenesis factors controlling appressorium development in pathogenic fungi. The identification and the characterization of *GUN1*, the ortholog of M. oryzae *Pth2* represents a proof of concept. Indeed, we show here that appressorium development is delayed by 1 day in the hypomorphic *gun1^SG^* mutant and by 2 days in the *Δgun1* mutant, demonstrating that, similarly to Pth2, GUN1 is also involved in appressorium functioning in *P. anserina*. The characterization of the other *GUN* mutants and, in the future, the isolation of new *GUN* mutants will be of great interest for better understanding ascospore germination as well as discovering new pathogenic factors, killing two birds with the same stone.

## MATERIALS AND METHODS

### Strains and culture conditions.

The strains used in this study are all listed in Table 8. All of these *P. anserina* strains derive from the wild-type *S* strain, ensuring a homogeneous genetic background (66, 67). The *Papks1^193^* mutant for the polyketide synthase encoding gene acting at the first step of melanin synthesis is described elsewhere (23). Standard culture conditions, media, and genetic methods for *P. anserina* were described previously (68) and can be found in the data of Silar (7) and on the *Podospora* database (http://podospora.i2bc.paris-saclay.fr/). The compositions of the M0 and M3 media are similar to that of the M2 medium, except that dextrin is replaced by glucose in the M3 medium (5.5 g L^−1^), while no carbon source is added in the M0 medium. This M0 medium was used as a basis for the development of media in which the only carbon source was sodium acetate (60 mM) or oleic acid (Sigma-Aldrich) (6 mM) dissolved in Tween 40 (0.5%). A control medium M0 with only Tween 40 (0.5%) was also used. The germination medium (CH_3_COONH_4_, 4.4 g L^−1^; Bacto peptone, 15 g L^−1^) used in this study was supplemented with yeast extract (G+YE) at 5 g L^−1^. In order to allow germination in strains producing ascospores unable to germinate, crosses were set up on M2 medium supplemented, when required, with tricyclazole (1 μg mL^−1^), a fungicide impairing melanin synthesis in *P. anserina* ascospores (23).

**TABLE 8:** Strains list

Strain	Genotype	Marker	Reference
*Wild-Type*	*big “S”*	none	68
*Δmus51*	*Δmus51::phleoR*	phleoR	
*Δmus51*	*Δmus51::genR*	genR	
*Δmus52*	*Δmus52::genR*	genR	
*PaMKK2^c^*	*PaMKK2^c^-phleoR*	phleoR	17
*ΔPaMpk2*	*PaMpk2::hygR*	hygR	17
*ΔPaNox2*	*PaNox2::phleoR*	phleoR	8
*PaPls1*	*PaPls1::hygR*	hygR	10
*Papks1^193^*	*Papks1^193^*	none	23
*Papks1^136^*	*Papks1^136^*	none	23
*mito-GFP*	*mito-GFP-hygR*	hygR	59
*GFP-SKL*	*GFP-SKL-phleoR*	phleoR	58
*Δpex5 Δpex7*	*Δpex5::hygR Δpex7::phleoR*	hygR, phleoR	29
*Δpex7*	*Δpex7::phleoR*	phleoR	29
*Δpex13*	*Δpex13::hygR*	hygR	33
*gun1^SG^*	*gun1^I441N^*	none	This study
*gun1^SG^ pGUN1*	*gun1^SG^ pBC-GUN1-genR*	genR	This study
*Δgun1*	*ΔPa_6_1340::hygR*	hygR	This study
*Δgun1 pGUN1*	*ΔPa_6_1340::hygR pBC-GUN1-genR*	hygR, phleoR	This study
*Δgup1*	*ΔPa_3_7660::phleoR*	phleoR	This study
*Δgup1 pGUP1*	*ΔPa_3_7660::phleoR pBC-GUP1-nouR*	phleoR, nouR	This study
*Δgun1 Δgup1*	*ΔPa_6_1340::hygR ΔPa_3_7660::phleoR*	hygR, phleoR	This study
*Δgun1 Δgup1 pGUN1*	*ΔPa_6_1340::hygR ΔPa_3_7660::phleoR pBC-GUN1-genR*	hygR, phleoR, genR	This study
*GUN1-mCherry*	*GUN1::mCherry-hygR*	hygR	This study
*GUN1-mCherry GFP-SKL*	*GUN1::mCherry-hygR GFP-SKL-phleoR*	hygR, phleoR	This study
*GUN1-mCherry mito-GFP*	*GUN1::mCherry-hygR mito-GFP-hygR*	hygR	This study
*GUN1-mCherry-AKI*	*GUN1::mCherry-AKI-nouR*	nouR	This study
*GUN1-mCherry-AKI GFP-SKL*	*GUN1::mCherry-AKI-nouR GFP-SKL-phleoR*	phleoR, nouR	This study
*GUN1-mCherry-AKI mito-GFP*	*GUN1::mCherry-AKI-nouR mito-GFP-hygR*	hygR, nouR	This study
*GUN1-mCHerry-AKI PaMKK2^C^*	*GUN1::mCHerry-AKI-nouR PaMKK2C-phleoR*	nouR, phleoR	This study
*gun1^SG^-mCherry*	*gun1^SG^::mCherry-hygR*	hygR	This study
*gun1^SG^-mCherry GFP-SKL*	*gun1^SG^::mCherry-hygR GFP-SKL-phleoR*	hygR, phleoR	This study
*gun1^SG^-mCherry mito-GFP*	*gun1^SG^::mCherry-hygR mito-GFP-hygR*	hygR	This study
*gun1^SG^-mCherry-AKI*	*gun1^SG^::mCherry-AKI-nourR*	nouR	This study
*gun1^SG^-mCherry-AKI GFP-SKL*	*gun1^SG^::mCherry-AKI-nouR GFP-SKL-phleoR*	phleoR, nouR	This study
*gun1^SG^-mCherry-AKI mito-GFP*	*gun1^SG^::mCherry-AKI-nouR mito-GFP-hygR*	hygR, nouR	This study
*gun1^SG^ Δpex7*	*gun1^SG^ Δpex7::phleoR*	phleoR	This study
*Papks1^136^ GUN1-mCherry mito-GFP*	*Papks1^136^ GUN1::mCherry-hygR mito-GFP-hygR*	hygR	This study
*Papks1^136^ GUN1-mCherry-AKI GFP-SKL*	*Papks1^136^ GUN1::mCherry-AKI-nouR GFP-SKL-phleoR*	phleoR, nouR	This study
*Papks1^136^ GUN1-mCherry-AKI mito-GFP*	*Papks1^136^ GUN1::mCherry-AKI-nouR mito-GFP::hygR*	hygR, nouR	This study
*Papks1^136^ GUN1-mCherry GFP-SKL*	*Papks1^136^ GUN1::mCherry-hygR GFP-SKL-phleoR*	hygR, phleoR	This study
*Papks1^136^ GUN1^SG^-mCherry GFP-SKL*	*Papks1^136^ gun1^SG^::mCherry-hygR GFP-SKL-phleoR*	hygR, phleoR	This study
*Papks1^136^ GUN1^SG^-mCherry mito-GFP*	*Papks1^136^ gun1^SG^::mCherry-hygR mito-GFP-hygR*	hygR	This study
*Papks1^136^ GUN1^SG^-mCherry-AKI GFP-SKL*	*Papks1^136^ gun1^SG^::mCherry-AKI-nouR GFP-SKL-phleoR*	phleoR, nouR	This study
*Papks1^136^ GUN1^SG^-mCherry-AKI mito-GFP*	*Papks1^136^ gun1^SG^::mCherry-AKI-nouR mito-GFP-hygR*	hygR, nouR	This study

### Genetic screening of constitutively germinating mutants.

It has been shown that wild-type *P. anserina* ascospores do not germinate on standard M2 medium and that the *ΔPaNox2* and *ΔPaPls1* mutant strains produce ascospores unable to germinate on all tested media (8, 10). To isolate mutants producing spontaneously germinating ascospores, UV mutagenesis was performed on self-fertile *mat–/mat+*, wild-type *S*, *ΔPaNox2*, and *ΔPaPls1* strains. The selection process of the germination mutants is summarized in Fig. S1. The mycelia of the strains mutagenized were fragmented (as described in “Mycelium Fragmentation and Strain Purification” below) and spread on M2 plates at 1,000 CFU. Shortly after UV exposure (UV 254 nm, 250 J/m^2^), followed by a 1-day culture in the dark to prevent repair by photoreactivation, the mutated strains were grown on standard M2 medium for 1 week until they developed mature ascospore-producing perithecia. In order to recover spontaneously germinating ascospores, M2 medium plates were put on top of the plates bearing perithecia, which allowed ascospores to be harvested in bulk on M2 medium. In *P. anserina*, most of the progeny is composed of heterokaryotic *mat+/mat–* ascospores leading to self-fertile mycelium upon germination (42). The thalli produced by the spontaneously germinating ascospores were incubated until they formed mature perithecia projecting their ascospores. Ascospores produced by these perithecia were individually collected with a needle and transplanted onto M2 medium. To ensure their independence, a single mutant (i.e., a single spontaneously germinating homokaryotic ascospore) per initial plate was selected for further analyses. For every mutant, progeny analysis of mutant × wild-type *S* crosses showed that a single mutated locus was responsible for the mutant phenotype (i.e., spontaneous germination of ascospores). For mutants recovered with the *ΔPaNox2* and *ΔPaPls1* strains, genetic analyses showed that the mutations enabling germination were unlinked to the *ΔPaNox2* and *ΔPaPls1* mutations, respectively. Homokaryotic *mat*– and *mat*+ mutant strains were isolated from the progenies, and homozygous mutant crosses were performed to check for the fertility/sterility of the mutants. For every isolated strain, microscopic observations were also performed to determine whether the ascospores germinated through the germination pore at the tip of the ascospore or through any other part of the ascospore (data not shown).

### Tetrad analysis in the *gun1^SG^* and in the *Δgun1* strains.

**(i) Demonstration of the recessivity of the *gun1^SG^* allele.** *P. anserina* produces mainly asci containing four heterokaryotic/dikaryotic ascospores, allowing nonordered tetrad analysis of first division segregation (FDS) asci and second division segregation (SDS) asci. More detailed explanations on tetrad analysis in *P. anserina* can be found in Grognet et al. (69). To determine the dominance/recessivity of the *gun1^SG^* allele, we crossed the *gun1^SG^* mutant with the WT. In 30 asci of the progeny, we found that 16 asci contained four heterokaryotic ascospores unable to germinate on M2 medium (4 [nongerminating] ascospores) and 14 asci contained different number of ascospores germinating spontaneously: 3 (nongerminating), 1 (germinating) or 2 (nongerminating), and 2 (germinating) ascospores. Above all, we never observed asci containing more than two ascospores germinating on M2 medium. Assuming some ascospore germination failure (due to their manipulation or incomplete penetrance of the *gun1^SG^* phenotype), especially the ones germinating spontaneously, we concluded that (i) asci of the first type were SDS asci containing four *gun1^SG^/GUN1* ascospores of the WT phenotype (nongerminating on M2 medium) and (ii) asci of the second type were FDS asci containing two *gun1^SG^/gun1^SG^* of the GUN (Germination UNcontrolled phenotype; germinating on M2 medium) and two *GUN1/GUN1* ascospores of the WT phenotype. The *gun1^SG^* allele was thus recessive against the wild-type *GUN1* allele (i.e., only *gun1^SG^/gun1^SG^* ascospores germinated on M2 medium but not the *gun1^SG^/GUN1* ones).

**(ii) Demonstration of the recessivity of the *ΔPa_6_1340*::*hygR* (*Δgun1*) allele.** The recessivity of the *ΔPa_6_1340*::*hygR* (*Δgun1*) phenotypes was tested by crossing *ΔPa_6_1340*::*hygR* with the wild type. We sowed 20 asci on G+YE germination medium, and we obtained (i) nine asci containing two (demelanized, nongerminating, hygR) and two (melanized, germinating, hygS) ascospores; these were identified as first division segregation (FDS) asci containing two *ΔPa_6_1340*::*hygR*/*ΔPa_6_1340*::*hygR* and two wild-type *Pa_6_1340^+^*/*Pa_6_1340^+^* ascospores, respectively. The eleven other asci contained four (melanized, germinating, hygR) ascospores which were identified as SDS asci composed of four *ΔPa_6_1340*::*hygR*/*Pa_6_1340^+^* ascospores. These data demonstrated that the phenotypes due to *ΔPa_6_1340*::*hygR* deletion were recessive and that SDS rate for the *Pa_6_1340 locus* was 55%.

**(iii) Complementation test between the *ΔPa_6_1340*::*hygR* (*Δgun1*) and the *gun1^SG^* alleles.** We crossed *gun1^SG^* with *ΔPa_6_1340*::*hygR*, and we reasoned that if *ΔPa_6_1340*::*hygR* and *gun1^SG^* are allelic, no functional complementation in heterokaryotic ascospores in SDS asci is expected for the spontaneous germination of *gun1^SG^*: *ΔPa_6_1340*::*hygR/gun1^SG^* ascospores germinate spontaneously on M2 medium. In contrast, if *gun1^SG^* and *ΔPa_6_1340*::*hygR* are not allelic, the theoretical genetic cross can be written *Pa_6_1340*::*hygR GUN1* × *Pa_6_1340^+^ gun1^SG^*, and several genetic combinations can be generated in SDS and in FDS asci. However, more importantly, none of these combinations can in theory lead to the spontaneous germination of more than two heterokaryotic ascospores. As shown above, concerning the *Pa_6_1340*::*hygR* locus, both kinds of asci were obtained in the F_1_ progeny: the SDS asci (54%; *n* = 50) were composed of four (hygR, melanized) ascospores, and the FDS asci were composed of two (melanized, hygS) and two (nongerminating, demelanized, hygR) ascospores. These asci were characterized by sowing them on G+YE and testing them for hygromycin B resistance. Functional complementation was tested by sowing 27 SDS asci on M2 medium supplemented with hygromycin B. Five types of asci were obtained ranging from four germinating ascospores to no germination of the four ascospores. We counted six asci with four germinating ascospores, seven asci with three germinating ascospores, four asci with two germinating ascospores, and seven asci with one germinating ascospore, and three asci showed no germination. Taking into account the partial penetrance of the *gun1^SG^* phenotype, we interpreted that three or four spontaneous germination on M2 medium plus hygromycin B in the same ascus could only occur if the four ascospores in the ascus were of the *ΔPa_6_1340*::*hygR/gun1^SG^* genotype. This led us to conclude that functional complementation does not occur in SDS asci, showing that *gun1^SG^* and *ΔPa_6_1340* are allelic. Eventually, this also demonstrated that *Pa_6_1340* was the gene mutated in the *gun1^SG^* mutant responsible for the spontaneous germination phenotype.

### *gun1^SG^* genome sequencing and analysis.

In a first step toward identifying the gene mutated in *gun1^SG^* through whole-genome sequencing, we backcrossed the mutant for five generations with the parental wild‐type *S* strain to eliminate any mutation unrelated to the mutant phenotype. The *gun1^SG^* genomic DNA was extracted as described previously (70). The genomic DNA was then subjected to complete sequencing using Illumina technology at the Imagif facility, Gif-sur-Yvette, France (CNRS, I2BC Sequencing Facility, https://www.i2bc.paris-saclay.fr/sequencing/). Custom‐made libraries had 300-bp inserts, and sequencing was 76‐bp paired end. Coverage was 80-fold. The sequence reads were then mapped onto the latest version of the reference genome of the *S* strain (69). Potential mutations were detected using SAMtools and bcftools on the Galaxy web server (https://usegalaxy.org/).

### Deletion of *GUN1* and *GUP1* and construction of the *Δgun1 Δgup1* strain.

**(i) *Δgun1* strain.** The deletion of *Pa_6_1340/GUN1* and its paralog *Pa_3_7660/GUP1* was performed using deletion cassettes made of two overlapping PCR fragments (see Fig. S4 and S6) (17). This method is based on the generation of two DNA PCR fragments carrying a resistance marker flanked by either 5′ or 3′ flanking sequences of the targeted gene. For *Pa_6_1340/GUN1*, we first amplified the 803-bp 5′ and 486-bp 3′ flanking regions of the *S* strain DNA by PCR with the primer pairs 1340_1/1340_2 and 1340_3/1340_4, respectively (see Table S2). At the same time, the hygromycin B resistance marker was amplified with 1340_MkF and 1340_MkR (see Table S2) from the pBC‐hygR vector (71). In a second PCR round, using the primers 1340_1 and 1340‐MkR and the primers 1340‐MkF and 1340_4, the resistance marker was fused with the 5′ and 3′ flanking regions, respectively. Both PCR products were used to transform a *Δmus51*::*phleoR* strain, in which the *mus51* gene involved in the NHEJ repair system is replaced by a phleomycin resistance gene (phleoR), allowing a high rate of homologous recombination (50). Two hygromycin B‐resistant (hygR) transformants were obtained. Each one was crossed with the wild‐type *S* strain. We observed in the progeny that homokaryotic (hygR) ascospores did not germinate. Consequently, crosses were performed on M2 medium supplemented with tricyclazole, leading to the spontaneous germination of ascospores (23). The (hygR) thalli coming from spontaneously germinating ascospores were selected on M2 medium supplemented with hygromycin B, fragmented and (hygR, phleoS) *ΔPa_6_1340*::*hygR* (*Δgun1*) homokaryotic mycelia of each mating type were isolated. Deletion of *Pa_6_1340* was verified by Southern blotting (see Fig. S4). Only one strain was selected for further analyses.

**(ii) *Δgup1* strain.** The same protocol was performed to produce the deletion cassettes for *Pa_3_7660/GUP1*. Using the primers pairs: 7660_F1/7660_R2 and 7660_R3/7660_R4 (see Table S2), the 1,104-bp 5′ and 1,011-bp 3′ *Pa_3_7660* flanking regions were PCR amplified, while the phleomycin resistance marker was amplified with the primers 7660_MkF and 7660_MkR (see Table S2) from a pBC-phleoR plasmid (71). In a second PCR round, using the primers 7660_F1 and 7660_MkR and the primers 7660_MkF and 7660_R4, the resistance marker was fused with the 5′ and 3′ flanking regions. Both PCR products were used to transform a *Δmus51*::*genR* strain. A total of 26 phleomycin-resistant (phleoR) transformants were obtained, and two independent (phleoR, genS) *Δgup1*::*phleoR* strains were selected from the progeny of a cross with the wild-type *S* strain (germination of *Δgup1*::*phleoR* ascospores was as the wild type). Deletion in these two independent strains was verified by Southern blotting (see Fig. S6). Only one strain was selected for further analyses.

**(iii) *Δgun1 Δgup1* strain.** To construct the *Δgun1 Δgup1* double mutant, we crossed a *Δgun1* strain with a *Δgup1* strain on M2 supplemented with tricyclazole, we selected (hygR, phleoR) mycelia from spontaneously germinating ascospores on M2 medium supplemented with hygromycin and phleomycin. These mycelia were fragmented, and homokaryotic (hygR, phleoR) *Δgun1 Δgup1* strains of each mating type were isolated.

### Plasmid constructions for complementation of *gun1^SG^*, *Δgun1*, and *Δgup1* strains.

**(i) Construction of pGUN1.** The *Pa_6_1340/GUN1* CDS, its 803-bp 5′ upstream and 486-bp 3′ downstream sequences were amplified by PCR from wild-type *S* genomic DNA using 1340_1 and 1340_4 primers (see Table S2). The PCR product obtained was cloned blunt end into pBC-genR plasmid carrying a Geneticin resistance marker digested by EcoRV to produce the pBC-GUN1-genR plasmid (renamed pGUN1 for the sake of simplicity). The insert was verified by sequencing (data not shown). This plasmid was used to transform the *Δgun1*::*hygR* deletion strain. Two (genR) transformants were obtained and checked for the restoration of wild-type phenotypes in ascospores. To that end, two of these transformants were crossed with the *Δgun1*::*hygR* strain. In the progeny of both crosses, (hygR, genR) melanized homokaryotic ascospores germinated (on G+YE germination medium), allowing us to purify *Δgun1 pGUN1 mat*+ and *mat*– homokaryotic strains and to show that wild-type *GUN1* complemented the *Δgun1* mutation. The (hygS, genR) *GUN1 pGUN1* homokaryotic ascospores were also isolated in the progeny. These ascospores germinated as the wild type. The pGUN1 was also used to transform the *gun1^SG^* mutant. Three (genR) transformants were obtained and crossed with *gun1^SG^* to assess restoration of wild-type germination in the progeny. For one transformant, we observed that *gun1^SG^ pGUN1* progeny showed wild-type ascospore germination, i.e., *gun1^SG^ pGUN1* spores did not germinate spontaneously on M2 medium but germinated on G+YE germination medium, showing that ectopic wild-type *GUN1* complemented the *gun1^SG^* mutant (Table 2).

**(ii) Construction of pGUP1.** The *Pa_3_7660/GUP1* CDS, its 1,104-bp 5′ upstream and 1,011-bp 3′ downstream sequences were amplified by PCR from wild-type *S* genomic DNA using the primers 7660_F1 and 7660_R4 (see Table S2). The PCR product obtained was cloned blunt-end into pBC-nouR (carrying a nourseothricin resistance marker) digested by EcoRV to produce the *pBC‐GUP1-nouR* plasmid (renamed pGUP1). The pGUP1 plasmid was used to transform the *Δgup1*::*phleoR* deletion strain. A total of 17 (nouR) transformants were obtained and checked for the restoration of growth on acetate (ace +). Fourteen of them were (ace +), showing that wild-type *GUP1* complemented *Δgup1* mutation. Two of these complemented transformants were crossed with the wild-type *S* strain and (phleoR nouR) *Δgup1 pGUP1*, as well as (phleoS nouR) *Δgup1 pGUP1* homokaryotic *mat*+ and *mat*– strains were purified.

### Plasmid construction for GUN1- and gun1^SG^-mCherry/mCherry-AKI tagging.

Two kinds of tagging were undertaken: one with the mCherry-AKI reporter protein, carrying the AKI PTS1 peroxisome targeting signal present in C terminus of GUN1, added in C terminus of the mCherry, and a second with the standard mCherry without the AKI PTS1 signal. To this end, we constructed plasmids allowing integration of the *mCherry-AKI* CDS or the *mCherry* CDS in 3′ (and in frame) of *GUN1* or of *gun1^SG^* CDS at the endogenous *GUN1* locus by homologous recombination (see Fig. S8). To achieve this, the 621-bp region upstream of the stop codon (but downstream of the mutation present in the *gun1^SG^* allele) was PCR amplified with primers 1340GFP_F2 and 1340GFP_R1 (see Table S2) designed to incorporate the ApaI and XhoI restriction sites in the sequence, respectively. The PCR product was cloned blunt end into the pBC-genR plasmid previously digested with EcoRV. The insert was then sequenced, digested with the restriction enzymes ApaI and XhoI, and gel purified to be finally cloned upstream of the mCherry CDS into the pBC-mCherry-hygR plasmid digested by ApaI and XhoI (see Table S2). This pBC-GUN1-mCherry-hygR plasmid (renamed pGUN1-mCherry for the sake of simplicity) was sequenced and transformed into both *Δmus52*::*genR* and *gun1^SG^ Δmus52*::*genR*, and (hygR) transformants were selected. We obtained 1 (hygR) transformant for *Δmus52*::*genR* and 2 (hygR) transformants for *gun1^SG^ Δmus52*::*genR*. Every transformant showed red fluorescence under the microscope. Correct *GUN1-mCherry* and *gun1^SG^-mCherry* gene fusions were verified by sequencing (data not shown). One transformant of each genotype was selected and crossed with the *S* strain to purify (hygR, genS) *GUN1-mCherry* and *gun1^SG^-mCherry* homokaryotic *mat*– and *mat*+ strains in the progeny. Finally, we observed that *GUN1-mCherry* ascospores germinated as the wild-type and that *gun1^SG^-mCherry* ascospores germinated spontaneously on M2 medium. To construct the pBC-GUN1-mCherry-AKI-nouR plasmid, we amplified by PCR the insert present in pGUN1-mCherry with the primers 1340GFP_F2 and mCH_AKIR1, the latter primer allowing addition of the AKI coding sequence at the end of the mCherry (see Fig. S8). This 1,341-bp PCR fragment was cloned blunt end into pBC-nouR digested with EcoRV to give the pBC-GUN1-mCherry-AKI-nouR plasmid (renamed pGUN1-mCherry-AKI). The construction was sequenced and transformed into the *Δmus52*::*genR* strain. We selected one (nouR) transformant showing red fluorescence under the microscope, and we crossed it with the wild-type *S* strain to purify (genS, nouR) *GUN1-mCHerry-AKI mat*+ and *mat*– strains. This *GUN1-mCHerry-AKI* strain showed red fluorescence under a microscope, and correct GUN1-mCHerry-AKI gene fusion was verified by sequencing (data not shown). This *GUN1-mCherry-AKI* strain was then crossed with the *gun1^SG^* mutant to generate the *gun1^SG^-mCherry-AKI* strain by meiotic recombination between the *gun1^SG^* mutation and the mCherry-AKI-nouR insertion. In the progeny of this cross, we isolated 13 thalli from spontaneously germinating (nouR) ascospores on M2 medium supplemented with nourseothricin. All of these isolates were heterokaryotic self-fertile (*mat+/mat–*). Since the *gun1^SG^* mutation is recessive, we hypothesized that these isolates arose from *gun1^SG^-mCherry-AKI/gun1^SG^-mCherry-AKI* recombinant heterokaryotic ascospores. One of these isolates was fragmented, and (nouR) homokaryotic *gun1^SG^-mCherry-AKI* ascospores of each mating type were purified establishing the *gun1^SG^-mCherry-AKI* strain. Red fluorescence in this final strain was verified, and the presence of the *gun1^SG^* mutation, as well as correct *mCherry-AKI* integration, was verified by sequencing (data not shown). Germination of *gun1^SG^-mCherry-AKI* ascospores was spontaneous on M2 medium as for the *gun1^SG^* mutant.

### Mycelium fragmentation and strain purification.

For strains carrying the *Δgun1* deletion and which therefore cannot germinate, homozygous crosses were performed on M2 medium supplemented with tricyclazole (1 μg mL^−1^), leading to the spontaneous germination of ascospores. The purification of homokaryotic strains was performed through mycelium fragmentation as follows. A small implant of the thallus of interest (0.5 cm^2^) was set in a 2-mL tube containing 500 μL of H_2_O and ground using a FastPrep (TeSeE; Bio-Rad, Hercules, CA) for 20 s at 5,000 rpm. Then, 100 μL of the fragmented mycelium was spread on an agar plate, and small hyphal fragments were isolated using a stereomicroscope (magnification, ×40). These isolates were then placed on M2 medium and cultured for 2 days at 27°C. Homokaryotic (*mat+* or *mat–*) versus self-fertile heterokaryotic (*mat+*/*mat–*) genotypes of isolates were determined through a *mat*-type test using wild-type *S mat+* and *mat–* strains.

### Fertility assay.

Fertility was assayed by generating self-fertile *mat+/mat–* heterokaryons of the tested strains. To this end, implants (0.5 cm^2^) of each mating type of the strains of interest were placed in 2-mL tubes containing 500 μL of H_2_O and ground using a FastPrep (TeSeE) for 20 s at 5,000 rpm. Next, 10 μL of each heterokaryon was dropped onto M2 medium and cultured for 10 days at 27°C with light. A qualitative evaluation of perithecium formation directly on the plates and of ascospore production projected on the petri plate lids during 4 days was performed. Every heterokaryon were analyzed in duplicate.

### Quantification of ascospore production.

At day 9 (ascospore projection starts at day 7), for the homozygous crosses—WT × WT (WT), *Δgun1* × *Δgun1* (*Δgun1*), and *Δgun1 pGUN1* × *Δgun1 pGUN1* (*Δgun1 pGUN1*)—ascospores projected for 30 min on circle surfaces 2 cm in diameter were counted under a stereomicroscope. Eleven measures were made for each cross (*n* = 11). A Student statistical *t* test was performed to compare the three measures.

### Cellophane penetration assay.

An implant of each tested strain was placed on a cellophane layer (Bio-Rad). After 2, 3, 4, and 5 days of growth at 27°C, the cellophane layer was removed, and the presence of mycelium in the medium checked with a stereomicroscope to determine whether the strain had breached the cellophane layer. In parallel, the presence or absence and the morphology of appressoria in the cellophane layer were observed under a microscope as described previously (9, 72) (see Fig. S3).

### Microscopic observations.

Ascospores observations were performed in Ibidi eight-well chamber microslides (Gräfelfing, Germany). Each well was filled with 200 μL of either M2 or modified G liquid medium: the quantity of Bacto peptone was divided by 2 compared to standard G medium in order to reduce the fluorescing background noise due to Bacto peptone. Germination induction of this modified medium was not altered (data not shown). Crosses were performed on standard M2 medium. Once perithecia were mature, agar plugs bearing the perithecia were cut and placed above the microscopic chambers upside-down. Perithecia were left to project their ascospores into the well for 5 h. For mycelium observations, small squares of medium (1 cm^2^) with grown mycelium on them were cut at the edge of the thallus and placed upside-down in water on the coverslip of a microscopic chamber. Images were taken with an inverted microscope Zeiss spinning disk CSU-X1 (Oberkochen, Germany) using four lasers (405, 488, 561, and 640 nm) for fluorescence observations, their associated filters, and a sCMOS PRIME95 (Photometrics) camera at the Imagoseine Imaging Facility (https://www.ijm.fr/plateformes-et-services/plateformes/imagoseine/). The images were analyzed with Fiji (73).

### Colocalization quantification.

For every genotype analyzed, the Pearson’s correlation coefficient (PCC) was calculated in the mycelium and in ascospores using the Coloc 2 tool of the Fiji software. PCC estimates colocalization (codistribution) of both fluorescent signals (green and red). PCC values range from 1 for two images whose fluorescence signals are perfectly linearly related to −1 for two images whose fluorescence signals are perfectly, but inversely, related to one another, with intermediate values indicating partial colocalization. Values near zero reflect distributions of signals that are uncorrelated with one another (60). For mycelium analysis, from four to seven different hyphae (of different images) were analyzed. In hyphae, PCCs have been measured on ~20-μm regions of interest (ROIs) on one focal plan for a total number of measures ranging from 12 to 25 per genotype. The numbers of analyzed ROIs for each genotype are indicated in Fig. 5C. A Student *t* test was applied to compare the PCCs of the different genotypes analyzed. For ascospore analyses, the whole surface of one focal plan per ascospore was divided in several ROIs. The sizes and numbers (ranging from four to eight per ascospore) of ROIs were designed depending on the presence or not of lipid droplets, so that the latter could be excluded. For the mCherry-AKI tagging experiment (Fig. 6), the number of analyzed ascospores ranged from four to seven for a total number of PCC measures ranging from 21 to 34 per genotype and condition. These data are indicated in Fig. 6C. In order to account for the within-unit correlation induced by repeated observations on the same ascospores, a mixed-effect linear model was used to assess the statistical significance of the medium and strain on quantified values. A total of 21 statistical units were considered in the GFP-SKL experiment, and 19 statistical units in the case of mito-GFP. *Post hoc* pairwise comparisons were performed using a Bonferroni-corrected *t* test with the Satterwaithe approximation (unequal variances), considering a family-wise error rate of 5%. For the mCherry tagging experiment (see Fig. S9), the number of analyzed ascospores ranged from two to three for a total number of analyzed ROIs ranging from 10 to 15. A Student *t* test was used to assess the pooled values per genotype to compare the PCCs.

### Phylogenetic analysis.

Fungal genes homologous to *GUN1* were searched by using BLAST at the GenBank and MycoCosm databases (74, 75), using the default parameters with the GUN1 protein sequence as a query. For a selection of *Ascomycota*, *Basidiomycota*, and *Mucoromycota* species, hits with an E value lower than 10^−5^ were selected. Research for other homologs was carried out for each selected species on OrthoDB (51). The alignment was performed with MAFFT (76) and manually refined using Jalview (77). A phylogenetic tree was built using the maximum-likelihood method (PhyML 3.1 software using the default parameters) (78). The tree was visualized on the iTOL server (79). Bootstrap values of 100 replicates are indicated (see Fig. S5). The GUN1 I441 residue conservation was assessed by visualizing the alignment on Jalview (see Fig. S7) (77).

### Bioinformatic analysis of the GUN1 protein.

The analysis of the GUN1 protein sequence was carried out using multiple tools: CD-search, https://www.ncbi.nlm.nih.gov/Structure/cdd/wrpsb.cgi (80); Interproscan, https://www.ebi.ac.uk/interpro/search/sequence/ (81); and Prosite, https://prosite.expasy.org/ (82). Prediction of its localization was realized using wolfPSORT (https://wolfpsort.hgc.jp/) (83). For research of a mitochondrial targeting signal (MTS), we also used MitoFates (http://mitf.cbrc.jp/MitoFates/cgi-bin/top.cgi) (84). 3D homology modeling was performed using Swiss model (https://swissmodel.expasy.org/) (85), and a 3D structure prediction of the GUN1 protein was achieved using the I-TASSER platform (https://zhanglab.ccmb.med.umich.edu/I-TASSER/) (86). The 3D structure of the murine CAT (PDB 2H3B) was used for comparison. The predicted effect of the missense I441N mutation in *gun1^SG^* on the protein structure and stability was assessed using the STRUM server (https://zhanglab.ccmb.med.umich.edu/STRUM/) and the PROVEAN online tool (http://provean.jcvi.org/index.php), respectively (54, 87).

### CAT activity assay.

**(i) Mycelium protein extraction.** Mycelia growing on M2 medium (2 confluent plates per strain) were harvested after 2 days; placed in 2-mL tubes, each containing a tungsten bead (diameter, 3 mm) and 1 mL of potassium phosphate buffer (pH 7.4) supplemented with 2 mM EDTA; and ground in a TissueLyser II apparatus (Qiagen, Hilden, Germany) at 30 rpm/s for 4 min at 4°C. The lysate was centrifuged at 4°C for 20 min at 17,000 × *g*, and the supernatant was separated from the pellet (cell debris). The protein concentration in the supernatant was measured by the spectrophotometric Bradford method (Sigma Chemical Co., St. Louis, MO).

**(ii) Ascospore protein extraction.** Each strain was crossed (in homozygous crossing) on at least five M2 medium plates. When perithecia were mature, the projected ascospores were collected on agar plates topped with a cellophane layer and pulled together in a 2-mL tube for each strain, each containing a tungsten bead (diameter, 3 mm). Immediately after harvesting, each tube was flash frozen in liquid nitrogen. Ascospores were dry-crushed in a TissueLyser II apparatus (Qiagen) at 30 rpm/s for 4 min at −80°C. Ascospores were maintained at –80°C in precooled TissueLyser blocks. The crushed ascospores were resuspended in 200 μL of potassium phosphate buffer (pH 7.4) supplemented with 2 mM EDTA. The lysate was centrifuged at 4°C for 20 min at 17,000 × *g*, and the supernatant was separated from the pellet (cell debris). The protein concentration in the supernatant was estimated by the spectrophotometric Bradford method (Sigma Chemical Co.).

**(iii) Carnitine-acetyltransferase assay.** CAT activity was assayed as described previously (88). The reaction was monitored spectrophotometrically at room temperature by following the release of CoA-SH from acetyl-CoA using the thiol reagent 5,5′-dithiobis-nitrobenzoic acid (DTNB; Sigma Chemical Co.). The reaction mixture contained 100 mM Tris-HCl buffer (pH 7.8), 0.05 mM acetyl-CoA (Sigma Chemical Co.), 0.1 mM DTNB, 22 mM dl-carnitine chloride (Sigma Chemical Co.), and protein extract in final volume of 1.0 mL. The reaction was initiated by adding a volume of the protein extract, and the increase in absorbance was monitored at 412 nm. CAT activities were determined by measuring the initial velocity of the CoA-SH production reaction and then reported as the activity ratio of the wild-type *S* strain. Standard deviations and statistical analyses were calculated from four to seven biological replicates. Eventually, CAT activities were compared using a Fisher-Pitman permutation test.

### Western blot analysis.

PaMpk2 phosphorylation was assessed as described previously (17). Ascospores produced by homozygous crosses of the *S* strain, the *PaMKK2^c^* mutant and the *gun1^SG^* mutant were harvested on agar plates topped with a cellophane layer to facilitate the ascospore harvesting process. For germination induction, a cellophane layer with wild-type ascospores was transferred on G+YE medium for 2 h. Once collected, ascospores were flash frozen in liquid nitrogen and then dry disrupted in a Micro-Dismembrator (Sartorius) at 2,600 rpm for 1 min at −80°C. Crushed ascospores were resuspended in Laemmli buffer, placed 5 min at 100°C, and then centrifuged for 15 min at 14,000 rpm. Samples were placed on a 1-mm-thick 15% SDS-PAGE gel and migrated for 3 h at 130 V, 25 mA/gel, and 25 W. The gel was then transferred onto a polyvinylidene difluoride membrane. Hybridization with the anti p44/p42 or anti-phospho p44/42 antibodies (Cell Signaling Technology), diluted to 1/1,000, was carried out overnight at 4°C. Hybridization with the second antibody coupled to peroxidase (GE Healthcare), diluted to 1/1,000, was carried out for 1 h at room temperature. For the revelation, an Immobilon chemiluminescence kit (Millipore) was used according to the supplier’s recommendations. A relative quantification of the ratio P-PaMk2/PaMk2 has been done using Fiji software. The mean pixel intensity of a defined ROI, including the band of interest, was measured for P-PaMpk2 and PaMpk2 signals, and the background intensity mean was subtracted. The ratio P-PaMk2/PaMk2 was then normalized with the “wild-type noninduced” ratio (ratio = 1) to give the induction fold compared to this reference condition (Fig. 7).
