# Supplementary material for: GUN Mutants: New Weapons To Unravel Ascospore Germination Regulation in the Model Fungus Podospora anserina
Source: Microbiol Spectr. 2023 Feb 14;11(2):e01461-22. doi: 10.1128/spectrum.01461-22 (PMC10100959; doi:10.1128/spectrum.01461-22)
Supplement: Supplemental file 2 — Supplemental material. Download spectrum.01461-22-s0002.pdf, PDF file, 6.5 MB [file spectrum.01461-22-s0002.pdf]

Figure S1: Genetic screening of constitutively germinating mutants of *P. anserina*

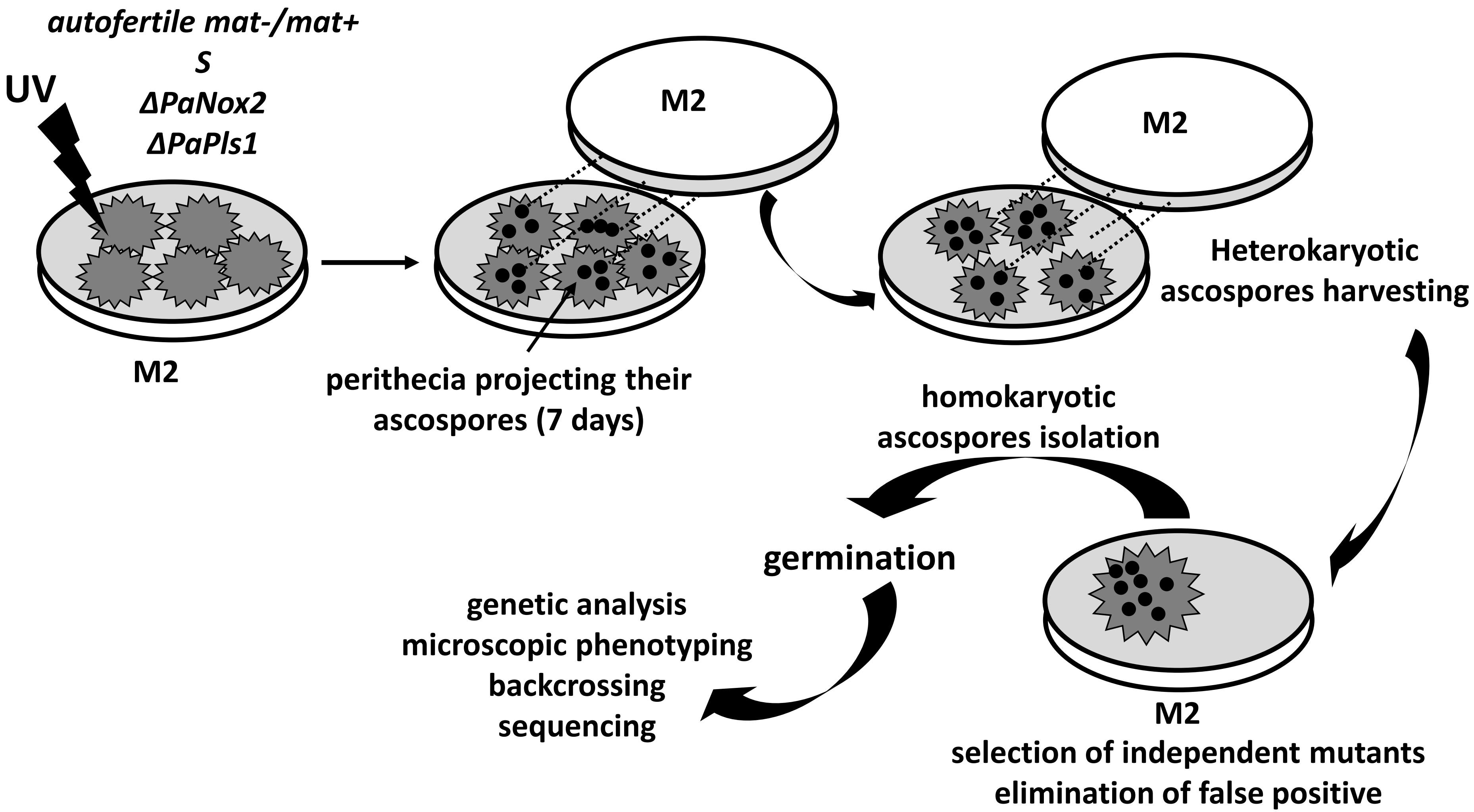

A

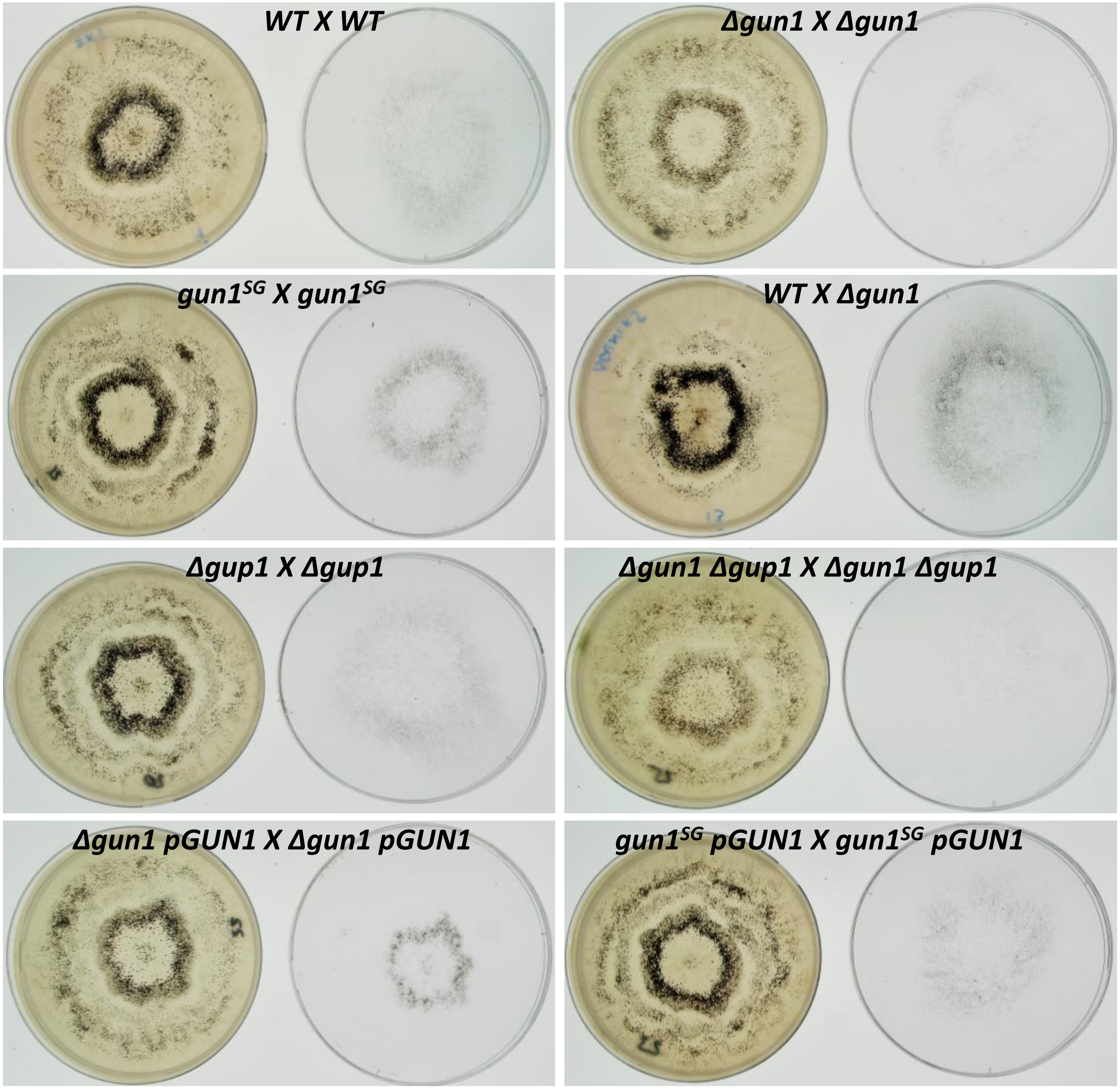

B

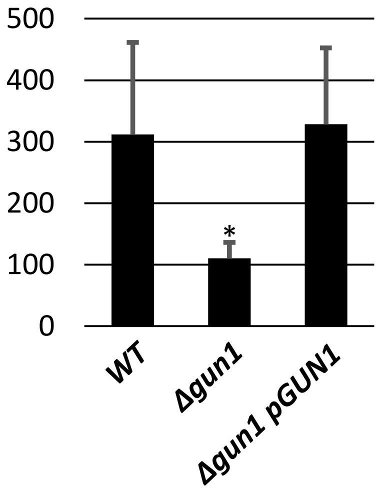

Figure S2: Fertility assays in heterokaryon crosses. A) A mix of *mat+* and *mat- mating type* of each genotype crossed is inoculated at the center of the petri plate and incubated 10 days at 27°C. Fertile perithecia project their ascospores from day 7 to day 10 directly on the lid of the petri plate. A slight reduction of the amount of perithecia in *Δgun1 X Δgun1* and *Δgun1 Δgup1 X Δgun1 Δgup1* crosses can be observed***.*** *Note* that *Δgun1 and Δgun1 Δgup1* ascospores are partially demelanized and are less visible on the picture. However, careful observation indicates that they are less numerous than for the other crosses. **B) Quantification of ascospore production** for the homozygous crosses *WT X WT (WT), Δgun1 X Δgun1 (Δgun1) & Δgun1 pGUN1 X Δgun1 pGUN1 (Δgun1 pGUN1). Ascospores projected during 30 min on circle surfaces of 2 cm diameter were counted (n= 11).* *, t-test analysis indicates that the number of ascospores projected in the *Δgun1 X Δgun1 cross is significantly lower than in the WT X WT and in the Δgun1 pGUN1 X Δgun1 pGUN1 crosses:* p-value < 0.001.

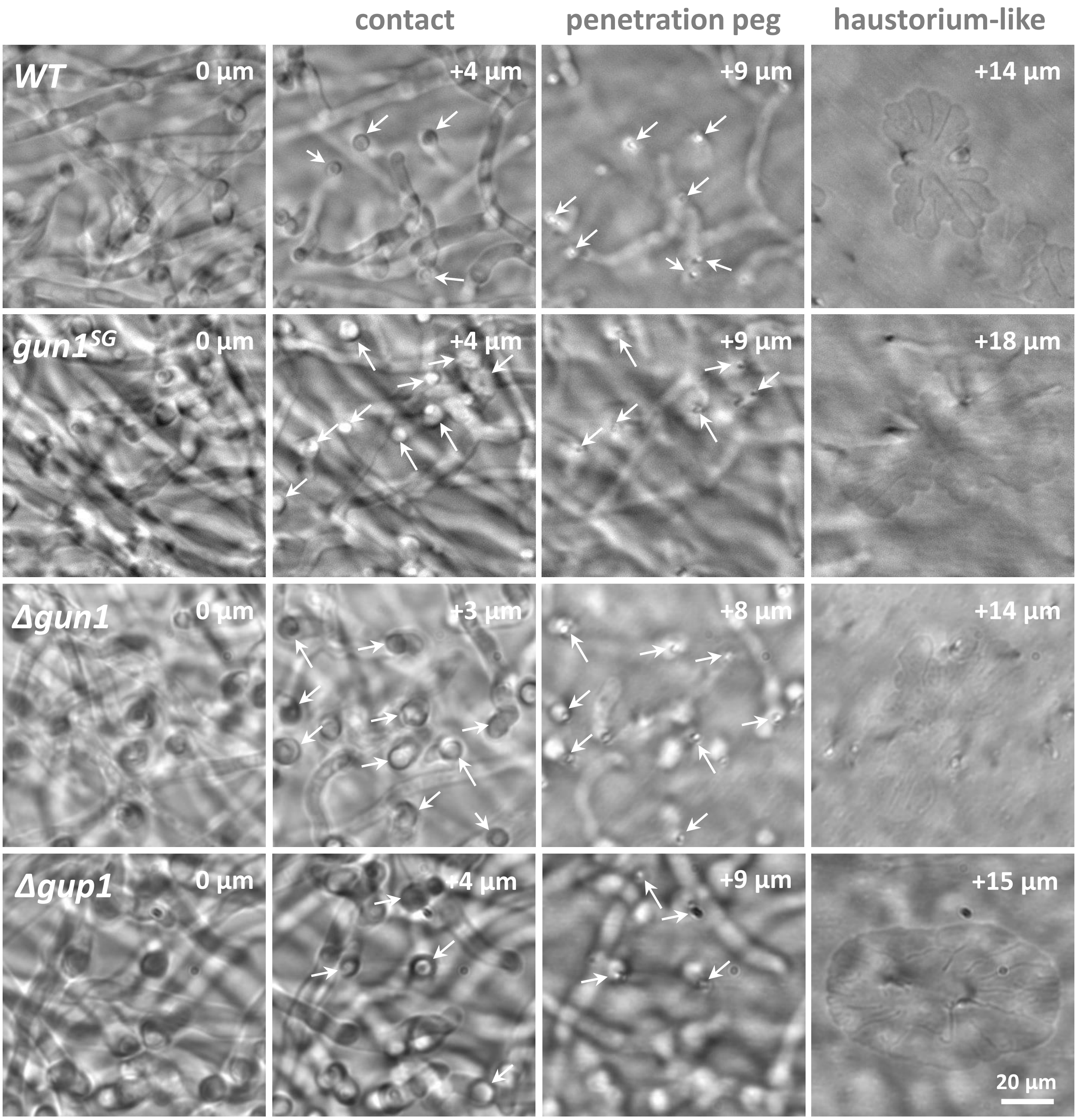

Figure S3: appressorium formation. Microscopic observations of four day-old mycelium from the indicated genotypes growing on a cellophane layer. From left to right, the different z-plans of the same field of view show the main steps of appressorium formation on cellophane: the reorientation of hyphal growth followed by the contact (arrows) of the hyphae with the cellophane layer, the formation of the penetration peg (arrows) and of the haustorium-like inside cellophane. The distances from the first picture are indicated in micrometres. Scale bar 20 µm.

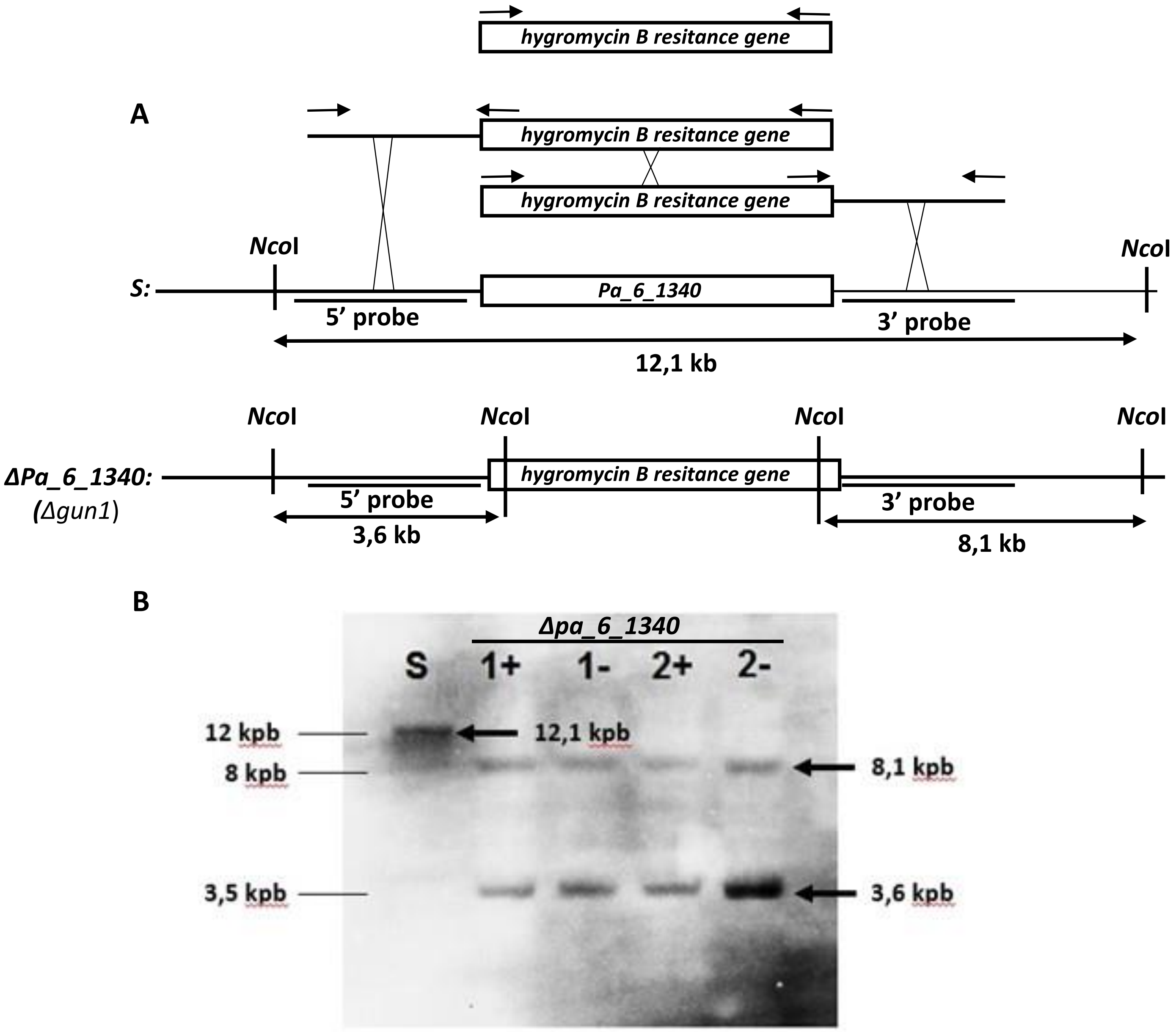

Figure S4: *Pa_6_1340/GUN1* deletion and Southern blot analysis. A, schematic representation of the PCR-based deletion strategy and the recombination events for homologous replacement of *Pa_6_1340* by the hygromycin B deletion cassettes. Restriction enzymes and positions of the probes used for Southern blotting are indicated. B, Southern blot analysis of the wild-type *S* strain (S) and transformants 1 and 2 in both mating types (+) *mat*+, (-) *mat*-. The sizes of the expected restriction fragments for the *S* strain and the deleted strains are indicated by an arrow.

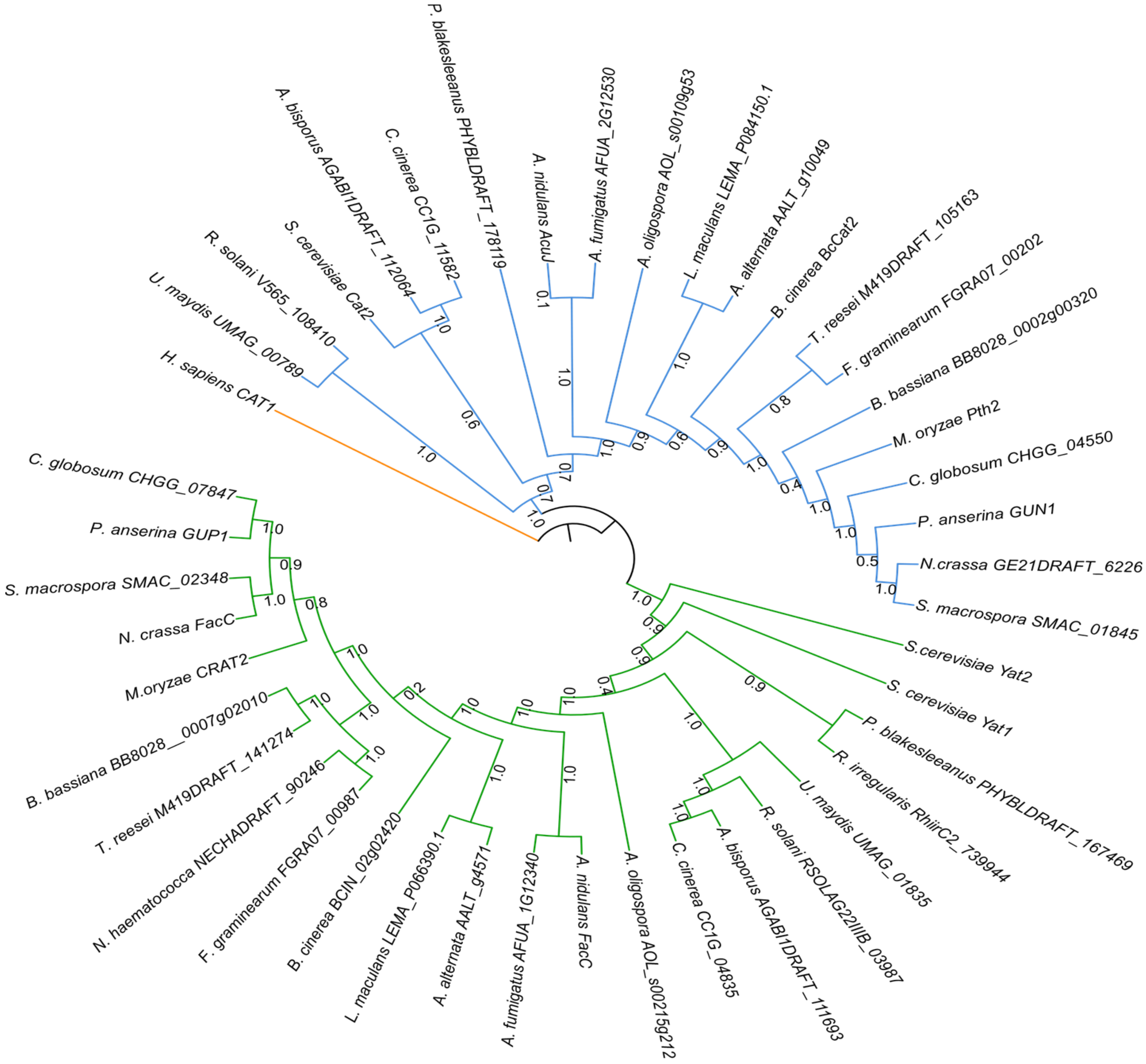

Figure S5: Maximum-likelihood phylogenetic tree of GUN1 homologues. The human homolog has been included to root the tree. Bootstrap values of 100 replicates are indicated. The blue branches correspond to the mitochondrial/peroxisomal-type CAT orthologs (GUN1, AcuJ, Pth2, etc.), the green branches correspond to the cytoplasmic-type CAT orthologs (GUP1, FacC, Crat2, etc.) and the orange branch corresponds to the out-group human CAT. **Analyzed species:** *Homo sapiens, Podospora anserina, Neurospora crassa, Magnaporthe oryzae, Aspergillus nidulans, Arthrobotrys oligospora, Botrytis cinerea, Ustilago maydis, Phycomyces blakesleeanus, Beauveria bassiana, Saccharomyces cerevisiae, Nectria haematococca, Trichoderma reesei, Chaetomium globosum, Penicillium chrysogenum, Leptosphaeria maculans, Sordaria macrospora, Fusarium graminearum, Rhizophagus irregularis, Alternaria alternata, Agaricus bisporus, Coprinopsis cinerea, Rhizoctonia solani , Aspergillus fumigatus.*

A

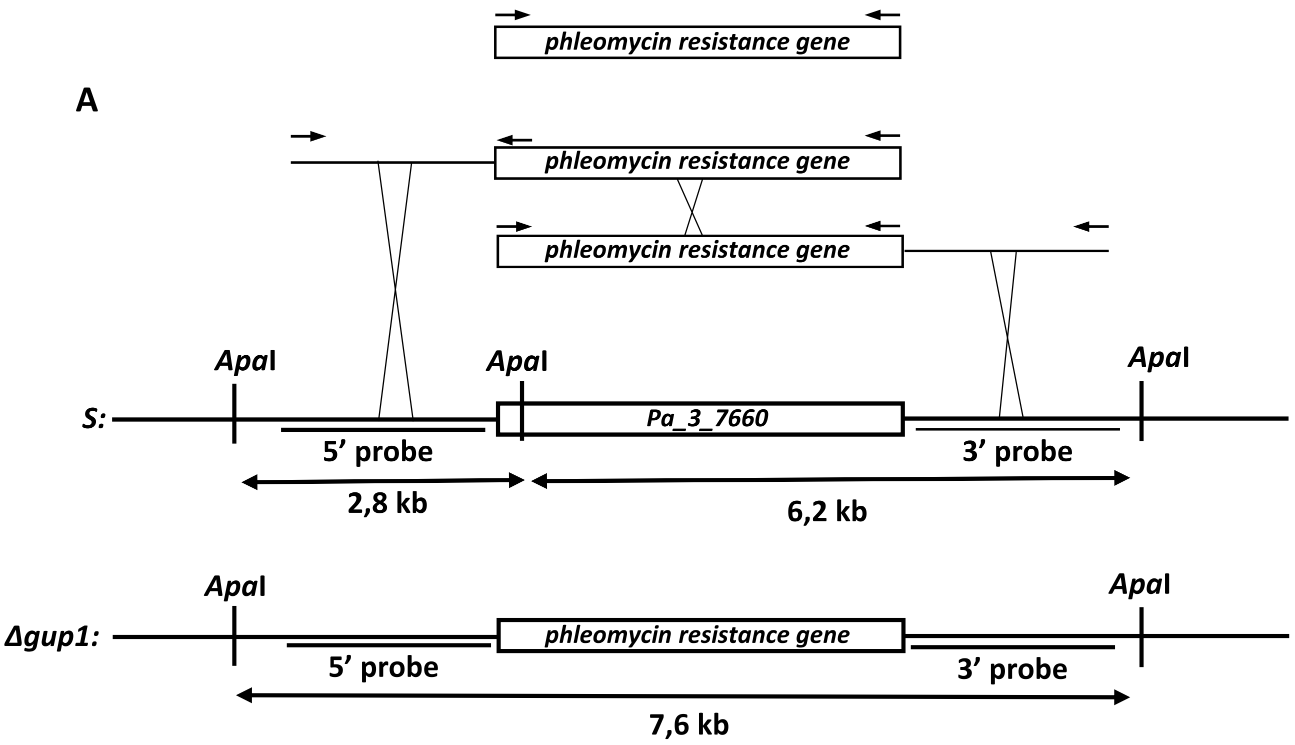

B

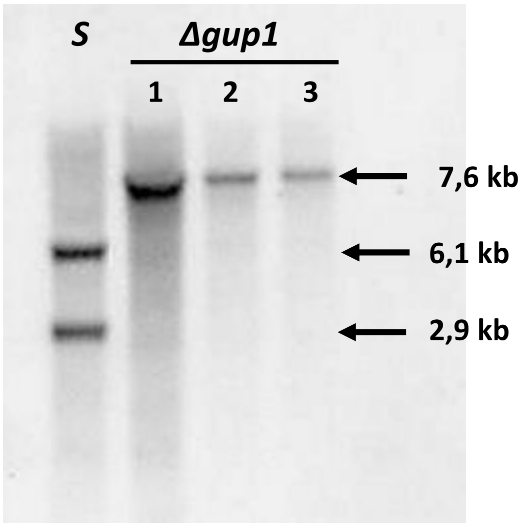

Figure S6: *Pa_3_7660/GUP1* deletion and Southern blot analysis. A, schematic representation of the PCR-based deletion strategy and the recombination events for homologous replacement of *Pa_3_7660* by the phleomycin deletion cassettes. Restriction enzymes and positions of the probes used for Southern blotting are indicated. B, Southern blot analysis of the wild-type *S* strain (S) and transformants 1, 2 and 3 (*mat*+). The sizes of the expected restriction fragments for the *S* strain and the deleted strains are indicated by an arrow.

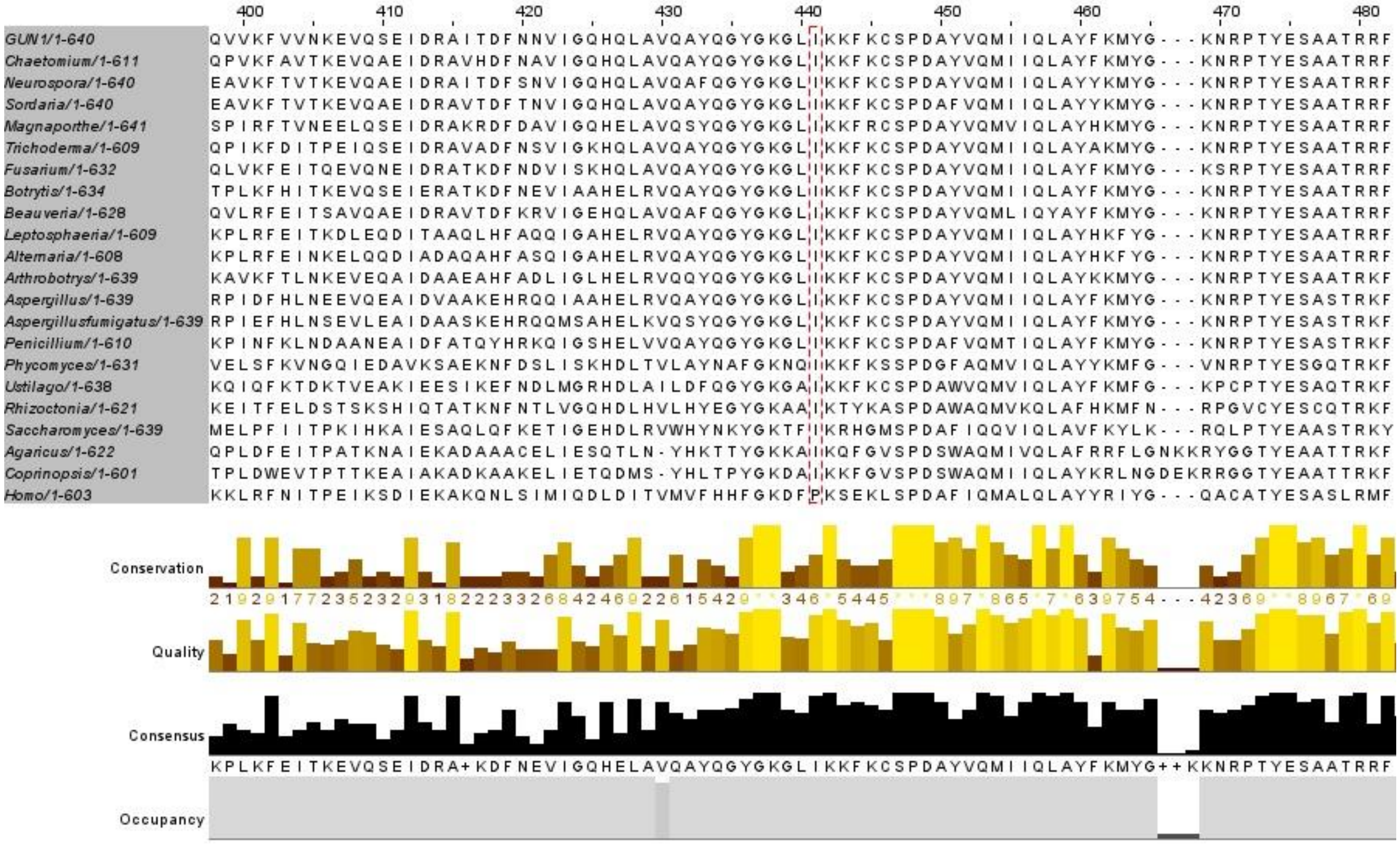

Figure S7: Alignment of GUN1 orthologues (from residue 398 to 482). The alignment was made with MAFFT and visualized with Jalview. The conserved residues are boxed in red. The sequence of the *H. sapiens* was used as non-fungal CAT sequence. The proline 422 of the human CAT is aligned with the isoleucine 441 of *P. anserina.* **Analyzed species:***, Podospora anserina, Chaetomium globosum, Neurospora crassa, Sordaria macrospora, Magnaporthe oryzae, Trichoderma reesei, Fusarium graminearum, Botrytis cinerea, Beauveria bassiana, Leptosphaeria maculans, Alternaria alternata, Arthrobotrys oligospora, Aspergillus nidulans, Aspergillus fumigatus, Penicillium chrysogenum, Phycomyces blakesleeanus, Ustilago maydis, Rhizoctonia solani, Saccharomyces cerevisiae, Agaricus bisporus, Coprinopsis cinerea, Homo sapiens.*

pGUN1-mCherry :

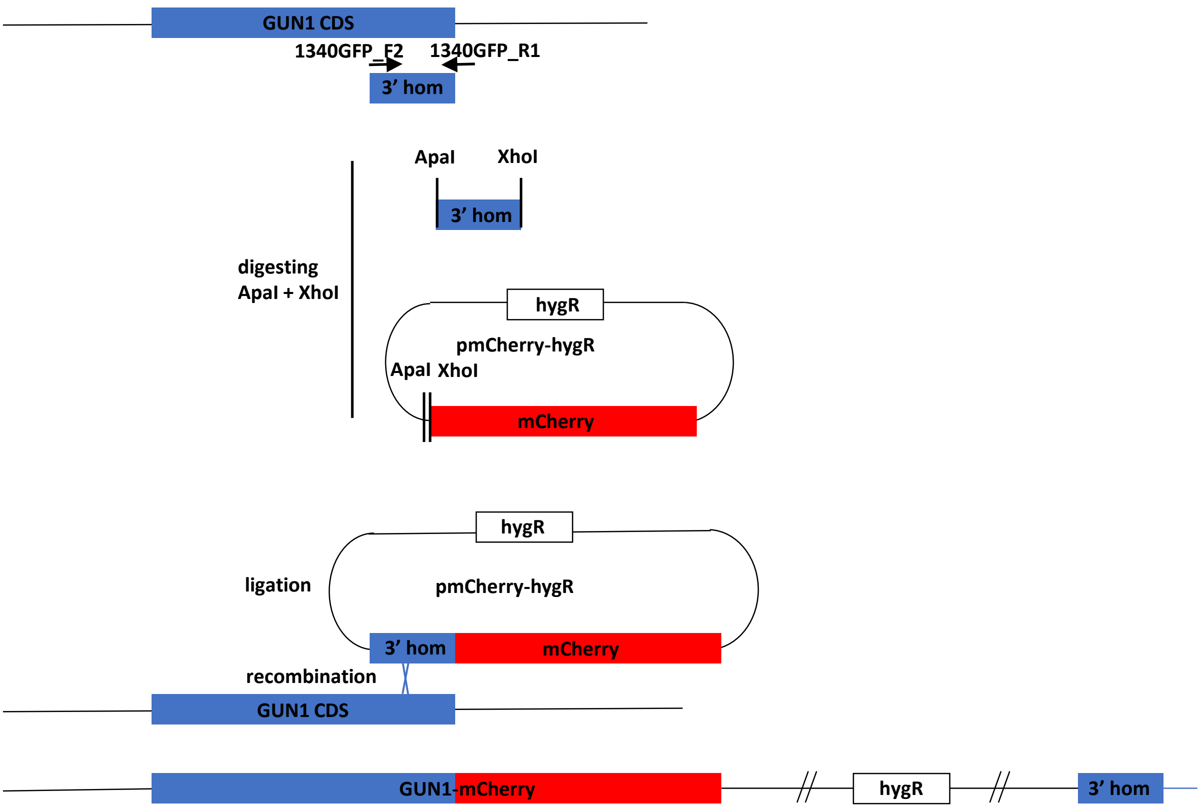

pGUN1-mCherry-AKI :

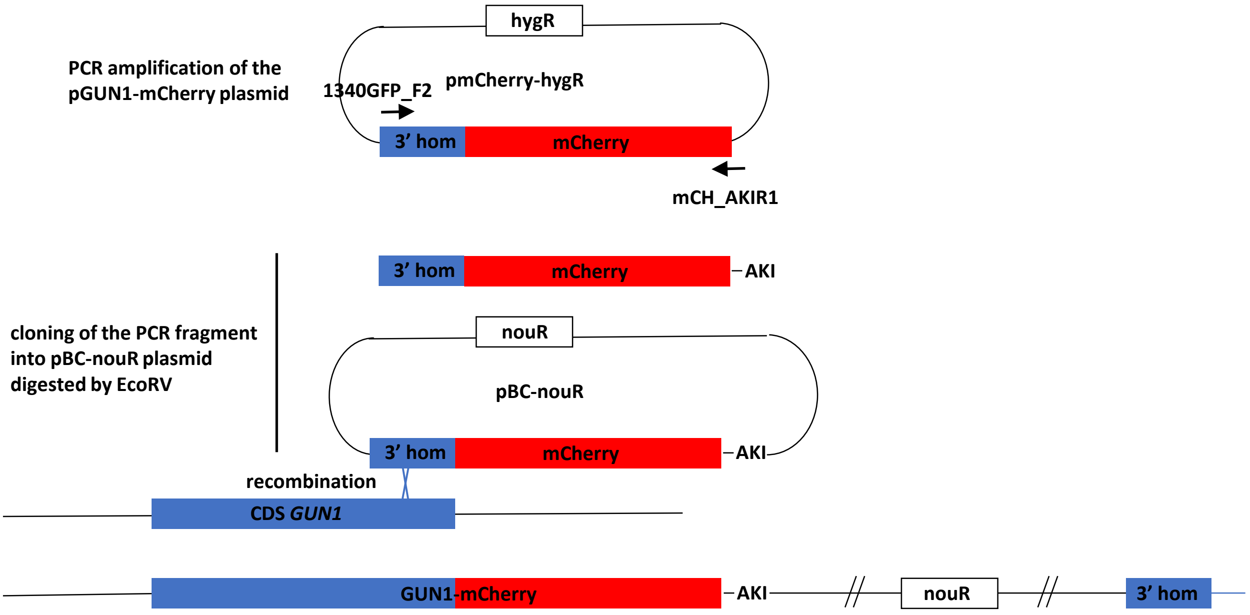

Figure S8. Schematic representation of the construction of the pGUN1-mCherry and pGUN1-mCherry-AKI plasmids

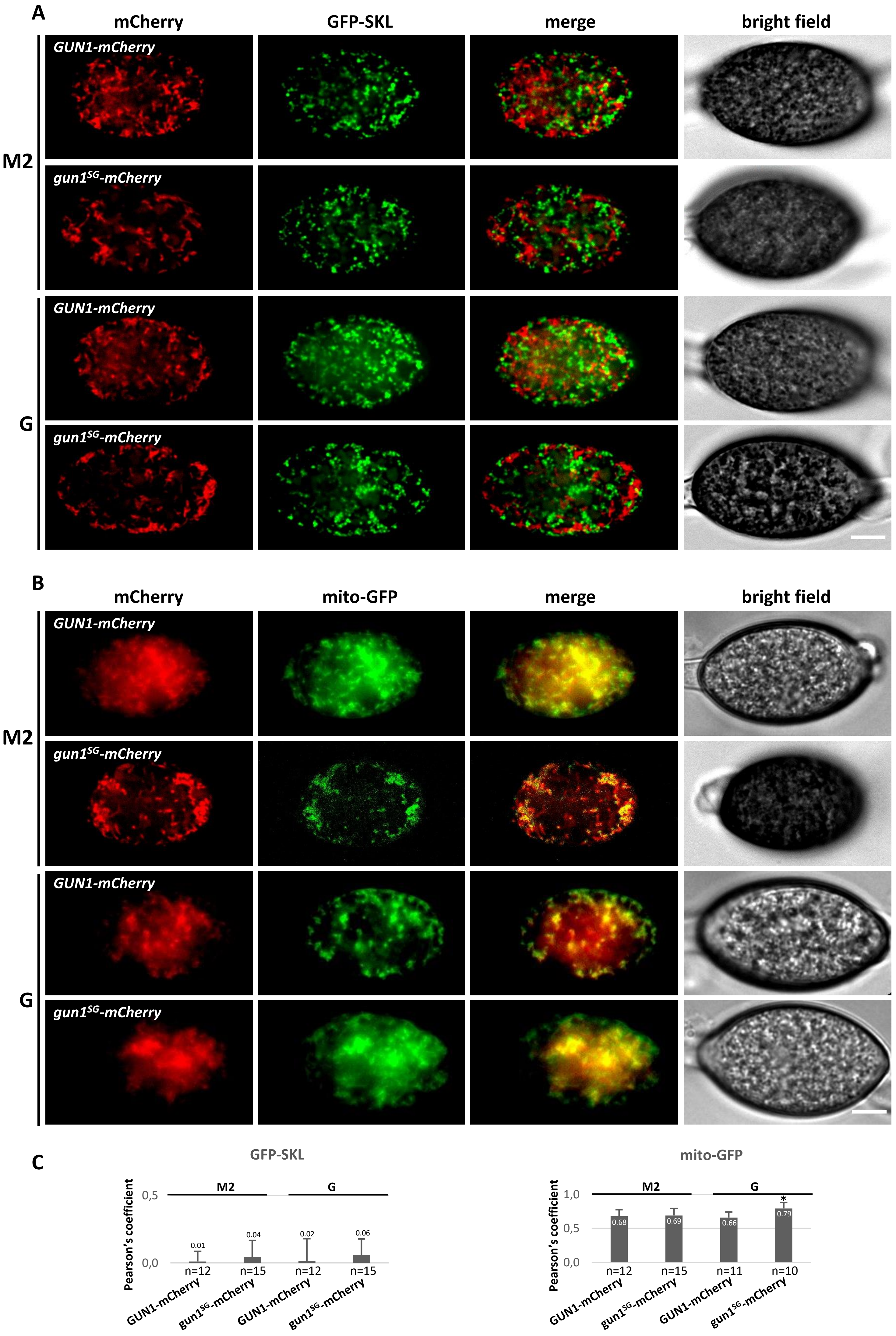

Figure S9: Spinning disc imaging of GUN1-mCherry and gun1^{SG}-mCherry in ascospores. All the ascospores imaged carry the *Papks1^{136}* mutation partially impairing ascospore melanisation making ascospores transparent for fluorescence imaging. A) Every strains carry the GFP-SKL reporter tagging peroxisomes. B) Every strains carry the mito-GFP reporter tagging mitochondria. M2 liquid medium: no induction of ascospores germination. G liquid medium: ascospores germination induction. Scale bar, 5 µm. C) Co-localization analysis: calculation of the Pearson's correlation coefficient. The number of analysed area per genotype is indicated. A T-test statistical analysis has been performed to compare the Pearson's coefficients calculated. *, statistically different from the three other genotypes analysed, p-value<0.05.

ascospores	black								pigmentless	
germination	through the primary appendage				through the primary appendage and the pore		through the pore first and then through the primary appendage	through the pore only	through the pore or any part of the spore	
mycelium	WT	WT	with sectors#	altered	WT	altered	flimsy	WT	pigmentless	pigmentless
Female fertility	WT		decreased	decreased	WT	sterile	sterile	WT	WT	WT
									not allelic with *PaPKS1*	allelic with *PaPKS1*
	SGD1<br>SGD5<br>SGD6<br>SGD7<br>SGD15<br>SGD18<br>SGD23<br>SGD40	SGD4<br>SGD12<br>SGD13<br>SGD26<br>SGD32<br>SGD646<br>SGD65	SGD2<br>SGD9<br>SGD16<br>SGD34<br>SGD975	SGD3<br>SGD56<br>SGD60<br>SGD62<br>SGD976	SGD21<br>SGD30<br>SGD36	SGD971	SGD22<br>SGD50<br>SGD51<br>SGD52<br>SGD53<br>SGD55<br>SGD57<br>SGD58<br>SGD59*<br>SGD61<br>SGD978	SGD27=GUN3<br>SGD28=GUN1<br>SGD29=GUN2<br>SGD31=GUN4<br>SGD35=GUN5<br>SGD39=GUN6	SGD25<br>SGD63<br>SGD644<br>SGD66	SGD8<br>SGD10<br>SGD14<br>SGD17<br>SGD19<br>SGD20<br>SGD33

Mutagenized strain: ***S***
ΔPaNox2
ΔPaPlas1

Table S1: Isolation of the Spontaneous Germination Defect mutants (SGD). Once the mutants were isolated, several phenotypes were analyzed: ascospore pigmentation, ascospore germination, mycelium aspect and female fertility (all the mutant isolated were male fertile). For the pigmentless ascospores, a cross with the *Papks1*^{193} mutant strain was performed in order to determine whether they were allelic with the pigmentless mutant *Papks1*^{193}. Altered: mycelium morphology different from the wild-type. *, leaky female sterility. # multiple sectors of flimsy growth.

PRIMER	SEQUENCE
pBC-For	5'-CGCGCGTAATACGACTCA-3'
pBC-Rev	5'-CGCGCAATTAACCCTCAC-3'
Fluo-MCS_For	5'-GGTTCCTGGCCTTTTGCT-3'
Fluo-MCS_Rev	5'-TCCTCGCCCTTGCTCAC-3'
1340_1	5'- CAAGAGGTGCGATTGGAGGAGGAGAGGG-3'
1340_2	5'-CTATTTAACGACCCTGCCCTGAACCGCCCAACTTCTCCTAGGCAAGTGGAGC-3'
1340_3	5'-CTTACCGCTGTTGAGATCCAGTTCGATGGCTCACGGTTGAGGGATCTAACGGGGGG-3'
1340_4	5'-GGGGGTAGGAAGAGAGGAGAGAAGTGGAG -3'
1340_MkF	5'-GCTCCACTTGCCTAGGAGAAGTTGGGCGGTTCAGGGCAGGGTCGTTAAATAG-3'
1340_MkR	5'-CCGTTAGATCCCTCAACCGTGAGCCATCGAACTGGATCTCAACAGCGGTAAG-3'
7660_F1	5'-GGCTGCCAATTGGTGGGGATCTAAACGTCA-3'
7660_R2	5'-CTATTTAACGACCCTGTGAACCGTTCTTGTCAGCTGGGGTGATCTGGATTCAA-3'
7660_F3	5'-CTTACCGCTGTTGAGATCATGGCTTGGCTATTGTGATGGGAGGGGGGGAGG-3'
7660_R4	5'-TATTTCTCAGGAAAGGTGGGGAGGCGGCCA-3'
7660_MkF	5'-TTGAATCCAGATCCAGCTGACAAGAACGGTTCAGGGCAGGGTCGTTAAATAG-3'
7660_MkR	5'-CCTCCCCCTCCCATCACAGCCAAGCCATCGAACTGGATCTCAACAGCGGTAAG-3'
Vérif 5'_1340	5'-GTCTGTCCGTTGCCTCCTTA-3'
Vérif 3'_1340	5'-TCCACCTTTTGCAGCCCCC-3'
Vérif 5'_7660	5'-CGTCTTCGTTTTGGTCTTTGTC-3'
Vérif 3'_7660	5'-ATGGGATTGGTGGTGGTTTG-3'
1340GFP_F2	5'-CTCGAGTACGTGCAGATGATCATCCAGCT-3'
1340GFP_R1	5'-GGGCCCCGATCTTGGCCTTGGGCGCCTCAA-3'
mCH_AKIR1	5'- TTAGATCTTGGCCTTGTACAGCTCGTCCATGCCGCCGG-3'
Valid_Mk_5'	5'- TGAGAAGCACACGGTCAC -3'
Valid_Mk_3'	5'- TCGGGGCGAAAACTCTC -3'
1340F3	5'- GCGTGGACAGGCATTTGT -3'
1340R2	5'-ACGCCGGATCCTGATACA-3'
7660_R5	5'-TGGATTAGAACACCTGGTGGAGGAATCGC-3'

Table S2: primers list

Strain	dextrin	acetate	Tween 40	oleic acid
GUN1-mCherry	+	+	+	-
gun1^{SG}-mCherry	+	+	+	-
GUN1-mCherry-AKI	+	+	+	+
gun1^{SG}-mCherry-AKI	+	+	+	+

Table S3: Growth of mCherry and mCherry-AKI tagged strains on different carbon sources. All the media have the same composition, except for the carbon source. The Tween 40 control medium and the oleic acid medium contain 0,5% Tween 40. +, wild-type growth; -, altered growth

GUN1-mCHerry-AKI **[nouR, pheloS]**	***PaMKK2^{C}*** **[nouS, pheloR]**	***+*** **[nouS, pheloS]**	***GUN1-mCHerry-AKI PaMKK2^{C}*** **[nouR, phleoR]**	**Total**
33	34	8	9	84

Table S4: Analysis of the genetic linkage between *PaMKK2^{c}* and the *GUN1* locus: *PaMKK2^{C}-phleoR* X *GUN1::mCHerry-AKI-nouR.* 123 homokaryotic ascospores were sown on G+YE medium. X^2 test performed on the genotype distribution determined that parental genotypes (*GUN1-mCHerry-AKI* and *PaMKK2^{C}*) were overrepresented, indicating genetic linkage between both loci analysed; X^2-test, p-value=1.5.10^{-6}. Recombination frequency, r=(8+9)/84=0.202; genetic distance, d=100*r=20.2 cM.
